# Circular RNA Calm4 Regulates Hypoxia-Induced Pulmonary Arterial Smooth Muscle Cells Pyroptosis via the Circ-Calm4/miR-124-3p/PDCD6 Axis

**DOI:** 10.1161/ATVBAHA.120.315525

**Published:** 2021-03-04

**Authors:** Yuan Jiang, Huiyu Liu, Hang Yu, Yang Zhou, Junting Zhang, Wei Xin, Yiying Li, Siyu He, Cui Ma, Xiaodong Zheng, Lixin Zhang, Xijuan Zhao, Bingxiang Wu, Chun Jiang, Daling Zhu

**Affiliations:** 1 Central Laboratory of Harbin Medical University, Daqing, China (Y.J., H.L., H.Y., J.Z., W.X., Y.L., S.H., C.M., X. Zheng, L.X., X. Zhao, D.Z.).; 2 Department of Pharmacology, the State-Province Key Laboratories of Biomedicine-Pharmaceutics of China, Key Laboratory of Cardiovascular Research, Ministry of Education, College of Pharmacy, Harbin Medical University, China (Y.J., H.L., H.Y., J.Z., W.X., Y.L., S.H., C.M., X. Zheng, L.Z., X. Zhao, D.Z.).; 3 Department of Medical Oncology, The Third Affiliated Hospital of Harbin Medical University, China (Y.Z.).; 4 The Second Affiliated Hospital of Harbin Medical University, Heilongjiang Province, China (B.X.).; 5 Department of Biology, Georgia State University, Atlanta, GA (C.J.).; 6 State Province Key Laboratories of Biomedicine-Pharmaceutics of China, Daqing (D.Z.).; 7 Key Laboratory of Cardiovascular Medicine Research, Ministry of Education, Harbin Medical University, China (D.Z.).

**Keywords:** fluorescence, hypertension, pulmonary, phenotype, pyroptosis, upregulation

## Abstract

**Objective:**

Circular RNAs are emerging as important regulators of pulmonary hypertension where pyroptosis plays a critical role. However, whether and how the circular RNAs regulate pyroptosis remained unexplored. Here, we show evidence for the involvement of a specific circular RNA known as circ-Calm4 in pulmonary hypertension and the underlying signaling pathway in pyroptosis.

**Approach and Results:**

Circ-Calm4 was upregulated in both mouse model of pulmonary hypertension in vivo and cultured smooth muscle cells in vitro. We performed immunoblotting, quantitative real-time PCR, LDH (lactate dehydrogenase) release assay, Annexin V-FITC/propidium iodide double staining, Hoechst 33342/propidium iodide fluorescence staining, and immunostaining to clarify the roles of circ-Calm4 in pulmonary arterial smooth muscle cell pyroptosis. Silencing the circ-Calm4 with its small-interfering RNA mitigated the upregulation of pyroptosis related phenotypes induced by hypoxia. Luciferase reporter assays confirmed that miR-124-3p suppressed the luciferase activity of the circ-Calm4 and RNA fluorescence in situ hybridization showed the colocalization of circ-Calm4 and miR-124-3p. The circ-Calm4 was found to act as a competitive endogenous RNA to regulate miR-124-3p. The pyroptosis-related alterations were all diminished with miR-124-3p in hypoxic pulmonary arterial smooth muscle cells. Inhibition of the gene targeted by miR-124-3p encoding the *Pdcd6* (programmed cell death protein 6) abrogated pyroptosis-related phenotypes under hypoxia stimulation.

**Conclusions:**

Our findings show a new signaling pathway, the circ-Calm4/miR-124-3p/*Pdcd6* axis was demonstrated in regulation of hypoxia-induced pyroptosis, which may potentially be useful for the design of therapeutic strategies for protecting the cellular functionality against pyroptosis as well as pulmonary hypertension.

## Abstract 2

Supplemental Digital Content is available in the text.

**Highlights**

- This study showed the role and mechanism of circ-Calm4 in in hypoxia-induced pyroptosis.
- Circ-Calm4 acted as a sponge for mir-124-3p to regulate pyroptosis.
- *Pdcd6* (programmed cell death protein 6) was identified as a crucial target of mir-124-3p in hypoxia-induced pyroptosis.
- We revealed a new signaling pathway of pyroptosis mediated by circ-Calm4/mir-124-3p/ *Pdcd6* axis in hypoxic PH.

Pulmonary hypertension (PH) is characterized by the continuous increase of pulmonary arterial pressure leading to right ventricular hypertrophy, right heart failure, and death.^1^ The pathogenesis of PH is complex and has not yet been fully determined. Abnormal pulmonary vascular endothelial function, pulmonary arteriole contraction, and pulmonary vascular remodeling lead to a fatal disease of right heart failure. These basic pathological changes are collectively related to basic pathophysiological processes in cells, such as proliferation, angiostenosis, cellular phenotype switching, and endothelial-mesenchymal transition.^2^ In addition, forms of programmed cell death, including apoptosis,^3^ and autophagy,^4^ as well as cellular necrosis,^5^ have been reported to be involved in the development of PH. Therefore, discovering new cellular processes and explaining the mechanisms will provide novel approaches for the diagnosis and treatment of this disease.

Pyroptosis, another form of programmed cell death, differs from apoptosis and necrosis.^6,7^ Pyroptosis can lead to rapid formation of pathological pores in the plasma membrane and disruption of the ionic gradients, which is accompanied by cell swelling and rupture.^8^ The NLRP3 (nucleotide-binding oligomerization segment-like receptor family 3) inflammasome is the key factor in pyroptosis^9^ and interacts with ASC (apoptosis-associated speck-like protein containing a caspase recruitment segment) to recruit procaspase-1.^10^ Inflammasomes are reported to regulate processing of Caspase-1, and activated Caspase-1 cleaves Gasdermin D (*Gsdmd*), resulting in the generation of the GSDMD N-terminal fragment (GSDMD-N). Following pathological pore formation and cell rupture, pyroptosis induces the release of the proinflammatory cytokines IL-1β (interleukin-1β) and IL-18 (interleukin-18).^11–13^ Moreover, pyroptosis has been reported to participate in the occurrence and development of infectious diseases, nervous system diseases, and atherosclerotic diseases and is considered to be an important pathological process in diseases with a high mortality rate. Our previous studies revealed that the levels of activated Caspase-1 and IL-18 products are increased in the model of hypoxic PH, suggesting that pyroptosis is associated with the pathogenesis of PH. Multiple regulatory factors participate in regulation of pyroptosis: Caspase-1 is activated by various inflammasome complexes in the innate immune response^14,15^ and Caspase-11/4/5 trigger pyroptosis upon the recognition of cytosolic lipopolysaccharide.^16^ Therefore, the discovery of the regulatory factors and mechanisms of pyroptosis is urgently needed.

Circular RNAs (circRNAs) are a unique class of noncoding RNAs. CircRNAs are produced by backsplicing of protein-coding gene exons, sometimes with introns, in eukaryotic cells.^17^ CircRNAs are present in both the nucleus and cytoplasm, are more stable than linear RNA, and are highly conserved.^18,19^ CircRNAs act by different mechanisms to regulate the gene and protein expression, such as by regulating microRNA (miRNA) target genes,^20^ regulating RBP-dependent functions,^21^ enhancing protein function,^22^ acting as protein scaffolds,^23^ recruiting proteins,^24^ and producing unique circRNA peptides.^25^ Based on their functional diversity, circRNAs may regulate signaling pathways and targets related to pyroptosis in pulmonary arterial smooth muscle cells (PASMCs), leading to PASMC pyroptosis and PH.

CircRNAs contain many miRNA response elements and miRNA-binding sites. CircRNAs reduce the number of miRNA molecules by competitively binding to miRNAs, resulting in upregulation of miRNA target genes. Increasing evidence shows that circRNAs are involved in the development of many diseases through a mechanism related to adsorbing miRNAs by acting as sponges.^26–28^ These studies reveal that circRNAs regulate the downstream targets of miRNAs via sponging miRNAs, a function involved in many disease processes.

Our previous study showed that the novel circRNA circ-Calm4 was upregulated in a mouse model of hypoxia-induced PH.^29^ We also proved that the expression of miR-124-3p was significantly downregulated in PASMCs treated with hypoxia. However, whether circ-Calm4 is involved in regulating the PASMC pyroptosis process and whether circ-Calm4 regulates pyroptosis by adsorbing the target miRNA miR-124-3p and controlling the expression of miR-124-3p target proteins in PH have not been elucidated. The objectives of our study were to investigate whether circ-Calm4 plays a role in hypoxia-induced pyroptosis and to identify the mechanisms underlying the role of this circRNA in PH.

## Materials and Methods

The data that support the findings of this study are available from the corresponding author upon reasonable request.

### Animals

Healthy male C57BL/6J mice weighing 20 to 25 g were purchased from the Experimental Animal Center of Harbin Medical University. To avoid the possible effects of estrogen on the PH, we used male mice to perform the study. Before the experiments, the mice were housed in an animal room and provided food and water for 10 days. All experimental procedures were performed in accordance with and were approved by the Institutional Animal Care and Use Committee of Harbin Medical University.

### Establishment of the Hypoxia Model

The model of hypoxia-induced PH was established by maintaining mice under hypoxic conditions (Fi,O_2_ 0.12) for 21 days in a normobaric environmental chamber, as described in a previous study.^30^ Prepare an aliquot of the vector at 10^10^–10^11^ genome equivalents in 20 to 30 μL in Hanks’ Balanced Salt Solution. Hanks’ Balanced Salt Solution for control and C57BL/6J mice inhaled AAV9 viral particles carrying circ-Calm4 short hairpin RNA (sh-circ-Calm4) or scrambled (sh-Scr) fragments via dropwise nasal instillation for 10 days before establishment of the hypoxia-induced PH model. The viral particles were constructed by GENECHEM (Shanghai, China). All experimental procedures were performed in accordance with and were approved by the Institutional Animal Care and Use Committee of Harbin Medical University.

Sh-circ-Calm4 target: 5′-GGCGCTTCCTACCAGAAAC-3′.

### Echocardiography

Mice were anesthetized with amobarbital (60 mg/kg, intraperitoneal injection) and then placed on a platform. Cardiac anatomic and functional parameters were evaluated by 2-dimensional transthoracic echocardiography using a Visual Sonic Ultrasound system (Vevo2100). The pulmonary arterial velocity time integral, pulmonary arterial preejection time, and pulmonary arterial ejection time were obtained from stable images.

### Hemodynamic Evaluation

A 1.2 French pressure catheter (Scisense, Inc) was connected to a Scisense FA-404 recorder. When the right jugular vein was exposed, the catheter was inserted into the vein and was advanced into the superior vena cava and finally into the right ventricular vein. The right ventricular systemic pressure was continuously recorded for 30 minutes. After measurement of the right ventricular systemic pressure, the thorax was opened, and the heart was dissected and weighed for calculation of the right ventricular hypertrophy index (RV/LV+S).

### Culture and Treatment of PASMCs

Healthy male C57BL/6J mice of 8 to 10 weeks were euthanized by dislocation of the neck. Mice were dissected pulmonary arterials and denuded the endothelial cells with an iron wire. Pulmonary arterial smooth muscle cells were isolated by Trypsin-type I collagen and Trypsin. PASMCs were cultured in DMEM containing 20% FBS at passages 2 to 4 and were used for further experiments. Cells were cultured in humidified air containing 5% CO_2_ at 37 °C for 24 hours. Then, cells were cultured under hypoxic conditions for 24 hours in a Tri-Gas incubator (Heal Force, Shanghai, China) providing 3% O_2_/5% CO_2_/92% N_2_.

### Western Blot Analysis

Samples were subjected to 12% SDS-PAGE and were then transferred to nitrocellulose filter membranes. Membranes were blocked in 5% skim milk at 25 °C for 1.5 hours and were then incubated with the primary antibodies. Anti-Caspase-1 (1 µg/mL; BOSTER), anti-NLRP3 (1 µg/mL; BOSTER), anti-IL-1β (2 µg/mL; BIOSS, Shanghai, China), anti-IL-18 (0.76 µg/mL; Proteintech), anti-ASC (2 µg/mL; BIOSS, Shanghai, China), anti-PDCD6 (programmed cell death protein 6; 0.734 µg/mL; Proteintech), anti-Cleaved Caspase-1(1 µg/mL;Cell Signaling Technology), and anti-Actin (0.22 µg/mL; Proteintech) antibodies were used in this study. After 4 washes with PBS, membranes were incubated and reacted with horseradish peroxidase-conjugated secondary antibodies. Immunoreactive bands were developed with enhanced chemiluminescence reagents (Amersham, United Kingdom).

### Small-Interfering RNA, miRNA Transfection

The miR-124-3p mimic, negative control miRNA (NC), and miR-124-3p antisense inhibitor (AMO-124-3p) were synthesized by GeneChem (Shanghai, China). Circ-Calm4 and *Pdcd6* small-interfering RNA (siRNA) and a scrambled RNA fragment used as a control were synthesized by RIBOBIO (Guangzhou, China). The overexpression vector for Circ-Calm4 was constructed by GENECHEM (Shanghai, China). The cells were seeded in 6-well plates, cultured for 24 hours, and transfected with Circ-Calm4-siRNA, *Pdcd6*-siRNA, and siRNA-NC using the Lipo2000 (Invitrogen, Carlsbad, CA) at a final concentration of 100 nM. PASMCs at a confluence of 60% to 70% were transfected with 10 µL of siRNA and 10 µL of the Lipofectamine 2000 siRNA Transfection Reagent, which were separately diluted in 100 μL of DMEM. The reagents were incubated separately for 5 minutes, then mixed and incubated at room temperature for 15 minutes. The siRNA-transfection reagent mixture was added directly to the cells. Six hours later, cells were placed in anoxic chamber (37 °C, 3% O_2_, 5% CO_2_; Tri-Gas Incubator, Shanghai, China) and then cultured for 24 hours and used in subsequent experiments. The cells were seeded in 6-well plates, cultured for 24 hours, and transfected with 100 nmol/L miR-124-3p or AMO-124-3p, with Lipo2000 (Invitrogen, Carlsbad, CA) following the manufacturer’s protocol.

The sequences were as follows:

mmu-miR-124-3p: 5′-UAA G GCA CGC GGU GA AU G CC-3′

5′-CA U UC AC CG CGU GC CU UAUU-3′

mmu-AMO-124-3p: 5′-GG CA UU CACC GC GUGC CU UA-3′.

mmu-miR-NC: 5′-UUC UCC GA AC GUG UCA CG UTT-3′

5′-AC GUGAC ACG UU CGGA GAA TT-3′

mmu-AMO-NC: 5′-C AGUA CUUU UGUG UA GUA CAA-3′.

si-circ-Calm4-1 target: 5′-TGG GCGCT TCCTA CCA GAA-3′

si-circ-Calm4-2 target: 5′-GGC GCT TCCTA CCAG AA AC-3′

si-circ-Calm4-3 target: 5′-CC TAC CAGA AAC CGCA GGA-3′

si-PDCD6-1 target: 5′-GC TT CCT GTG GAACGTCTT-3′

si-PDCD6-2 target: 5′-GG GAC AACT C TGG GA TGAT-3′

si-PDCD6-3 target: 5′-GG ATCAG GATG G CT GGATT-3′

### Circ-calm4 and lincalm4 Overexpression in PASMCs

Overexpression vector for circ-Calm4 was constructed. Using Calm4 exon 1, as a template, PCR was performed with

5′- ACG G G CCCTC TA GAC TCG AGCAGA AACC GC AG G AG CAGC AG-3′

And 5′-TTA A ACTT AA GCTTGG TAC CATA AGC TTG AT ATC GA AT TCCGATC-3′ to obtain a fragment of 981 bp, which was the gene sequence of circ-Calm4 and upstream intron cyclization component and downstream intron cyclization component. Sequences are shown below:

CAGAAACCG CAGGAGCAG CAGACTGACATCCA GTTGACACCAATCTCAG AAGCTTTGTAAGGTA CAGAGATGTCTC ACGGGTTTA CTAAGGAGGAGGTCGC TGAGTTCCAGG CAGCTTTCAATAG GTTTGACAAGA ATAAGGATGGCCA TATCAGCGTCGAGG AACTTGGAGATGTAATGA AGCAGCTGGGCA AGAACCTCCC AGAGAAGGA CCTGAAGGCTC TCATCTCCAAGTTGGA CACAGATGGTGAT GGCAAGATCAGCT TTGAAGAATTCT TGACAGCCATAGA GAAGTACAAGAA AGGGCACAGAG CAGGGGAGCTG CGGGCTGTGTT CAATGTCCTTG ACCAGAATGGT GATGGCTACATCACTG TGGATGAGCTCAAGG AAAGCTTGTCCAAG TTAGGCGAGTCCCT TTCCCAGGAGGAGC TGGAGGACATGAT CCGTGTGGCTGAC GTGGACCAAGATG GGAAGGTGAAATA TGAGGAGTTTGTGC GCCTCCACGTTGAA AATTGAGACTGCTG GTCACAAGCTCC AACTCGTCGTCTATC TGCCTGTCTCTGA CAGGCACTTGTG CCTTCTTGCGGA GCTCTGCTTTTGT GGTGGTGTTAAA AAGAAGAGAAGTC TGTGTGGTGTATGG AAACTATCTCATGA GTGAGGAAGACTT TATTGGCTCTGGA TGCTGTGCCATCTG GAGCTGCTCACT AGTCCCCTCTCTGC CTGCTGAGACT CTGGTCAGAG GATGGTTTCCT TGGGTGTGTCTCT CCTCTCCCTCTTGCT GTGGAATGGGC GCTTCCTAC. XhoI I and KpnI were jointly connected to expression vector CMV-MCS-SV40-Neomycin through double enzyme connection.

Overexpression vector for lincalm4 was constructed. Using *Mus musculus Calm4* transcript variant (GenBank: NM_020036) as a template, PCR was performed with

5′-ACG G GCC CTCT AGAC TCG A GCGC CAC CATGTCTC ACGG GTTTAC TAAG-3′ and 5′-TTAA AC TTA AGCT TGG TACC TCAATTTT CAA CGTGG AG GC GCAC-3′ to obtain a fragment of 494 bp, which was the gene sequence of *Calm4* and upstream and downstream recombination exchange arms. XhoI I and KpnI were jointly connected to expression vector CMV-MCS-SV40-Neomycin through double enzyme connection. The cells were seeded in 6-well plates, cultured for 24 hours, and transfected with 2 µg overexpression plasmid and control vector, with Lipo2000 (Invitrogen, Carlsbad, CA) following the manufacturer’s protocol. PASMCs were harvested for total protein purification and RNA isolation.

### Quantitative Real-Time PCR

Total RNA samples were extracted from cultured PASMCs and lung tissue using TRIZOL reagent (Invitrogen). Blood RNA samples in mice were collected from the abdominal aorta using TRIZOL reagent. RNA samples in medium were extracted and isolated from PASMCs medium according to the manufacturer’s protocol (BestBio, Shanghai, China). Cytoplasmic and nuclear RNAs were isolated using a PARISTM kit (Thermo Fisher Scientific, Scotts Valley, CA) according to the manufacturer’s protocol). For each sample, 500 ng of total RNA was converted to cDNA using a Superscript First-Strand cDNA Synthesis Kit (Invitrogen). To measure the mRNA expression levels of circ-Calm4, miR-124-3p, *Pdcd6*, *Caspase-1*, *Nlrp3*, *Asc*, *Il-18*, and *Il-1β*, real-time PCR was carried out in a LightCycler 480 II real-time PCR system (Roche, Germany) with SYBR Green I (Applied Biosystems). Actin was used as an internal control for mRNA quantification, and U6 was used as an internal control for miRNA quantification. Threshold cycle (Ct) was determined and relative mRNA and miRNA levels were calculated based on the Ct values. The data were analyzed using the 2^−^^ΔΔCT^ method.

The following primers were used in this study:

*Caspase-1* forward, 5′-ACACGTCTTGCCCTCATTATCT-3′ and

*Caspase-1* reverse, 5′-ATA ACCTTG GGCTTGTCTTTCA-3′;

*Nlrp3* forward, 5′-GTG GAGA TCC TAGGTTTCTCTG-3′ and

*Nlrp3* reverse, 5′-CAGGA TCTCATTC TCTT GGATC-3′;

*Asc* forward, 5′-CTTGT CA GGGGA TGAAC TCAAAA-3′ and

*Asc* reverse, 5′-GCCAT ACGACT CCAGATAG TAGC-3′;

*Il-18* forward, 5′-GGCC TGA CATCTTCTGC AACCTC-3′ and

*Il-18* reverse, 5′-TG ACG CAAG AGTCTT CTG AC ATGG-3′;

*Il-1β* forward, 5′-CCCT GC AGCTG GAG AGTGTG G-3′ and

*Il-1β* reverse, 5′-TGTG CTCTGC TTGAG AGGTGCT-3′;

circ-Calm4 forward, 5′-GA AGAGA AGTCTGTGT G GTGTATGG-3′ and

circ-Calm4 reverse, 5′-GCT CCTGC GGTTT CTGGTAG-3′;

Actin forward, 5′-CTGT CCCTGT ATGCCT CTG-3′ and

Actin reverse 5′-ATGTCA CGCAC GA TTTCC-3′; and

*Pdcd6* forward, 5′-TAA CTGA GGCGGAAG CATAGCC-3′ and

*Pdcd6* reverse 5′-TCC ACTC CTGTCTTTA TCA ACCC-3′.

*Calm4* forward, 5′-TG TCC AAGT TAGGCGAGTCC-3′ and

*Calm4* reverse, 5′-AGGC AGAT AGACG ACG AGTTG-3′.

### Luciferase Reporter Assays

HEK-293T cells were cultured in 24-well plates to 70% to 80% density before transfection. Circ-Calm4 and the *Pdcd6* 3′ untranslated region (3′UTR) containing the conserved miR-124-3p binding sites or mutated sequences were synthesized by GeneChem (Shanghai, China). Five hundred nanograms of plasmids containing wild-type Circ-calm4 (Circ-calm4-wt), mutant Circ-calm4 (Circ-calm4-mut), miR-124-3p and NC were transfected using Lipo2000 (Invitrogen, Carlsbad, CA). Then, 500 ng of plasmids containing wild-type *Pdcd6* (*Pdcd6*-wt) and mutant *Pdcd6* (*Pdcd6*-mut), miR-124-3p and NC were transfected using Lipo2000 (Invitrogen, Carlsbad, CA). After 24 hours of incubation, the firefly and Renilla luciferase activities were measured using a Dual Luciferase Reporter Assay System (Beyotime Biotechnology, Shanghai, China).

The sequences were as follows:

Circ-Calm4-wt: GGTGAAATATGAGGAG TTTGTGCGCCTCC ACGTTGAAAATTG AGACTGCTGGTC ACAAGCTCCAA CTCGTCGTCTAT CTGCCTGTCTCT GACAGGCACTT GTGCCTTCTTGC GGAGCTCTGCT TTTGTGGTGGTGTT AAAAAGAAGAGA AGTCTGTGTGGTG TATGGAAACTATCT CATGAGTGAGGAA GACTTTATTGGCTCTGGAT Circ-Calm4-mut: GGTGAAATATGAG GAGTTTGTGCG CCTCCACGTTGA AAATTGAGACTGCT GGTCA CAAGCTCCAAC TCGTCGTCTATCTGCCT GTCTCTGACAGGCA CTTTGTAAGGCTTGC GGAGCTCTGCTTTTG TGGTGGTGTTAAA AAGAAGAGAAGTCTG TGTGGTGTATGGAA ACTATCTCATGAGTGA GGAAGACTTTATTGGCT CTGGAT *Pdcd6*-wt: ATCATGTTCT TTT CTAGATGTCTC TAAGTCTATAG TTGCAATGCTTTT ATTAGCCAATAGAA TTTTAAAATAATATGGAACTTG CACAGAAGGC TTTTCATGTG CCTTACTTTTTA AAAAAAATTG GAATATGTAACA TAAGCAATAAA GTGATGATACAGACT GGTTGCCTAGTTTT GGCTGGTTTTTTG CTTTGTTTTGTTTTAAC *Pdcd6*-mut: ATCATGTTCTTT TCTAGATGT CTCTAAGTC TATAGTTGCA ATGCTTTTATT AGCCAATAGAAT TTTAAAATAATAT GGAACTTGCACAGA AGGCTTTTCATCACGG AAACTTTTTAAAAAA AATTGGAATATGT AACATAAGCAATA AAGTGATGATACA GACTGGTTGCCTA GTTTTGGCTGGTTT TTTGCTTTGTTTTGTTTTAAC

### RNA Fluorescence In Situ Hybridization

A fluorescence in situ hybridization (FISH) assay was performed in PASMCs. Cy3-labeled circ-Calm4 probes and FAM-labeled miR-124-3p probes were designed by GeneChem (Shanghai, China). The signals of the probes were detected by a Fluorescence In Situ Hybridization Kit (GeneChem Shanghai, China) according to the manufacturer’s instructions. PASMCs were fixed in 4% paraformaldehyde and then incubated with Cy3-labeled circ-Calm4 probes and FAM-labeled miR-124-3p probes. Mix overnight at 37 °C. The images were analyzed with a fluorescence microscope. U6 FISH Probe was used to image the nuclei, 18S FISH Probe was used to localize the cytoplasm, NC-probe was used as a control and DAPI (4,6-diamidino-2-phenylindole) was used to label the nuclei.

The primer and probe sequences were as follows:

mmu-circ-Calm4: TGCTCCT GCG GTTTCTGG TAGG AAGCG CCC ATTCC

mmu-miR-124-3p: GGC AT TCAC CGCGTG CCTTAC.

Colocalization scatter diagrams can describe the degree of colocalization intuitively and quantitatively. The ScatterJ^31^ plug-in in ImageJ^32^ were used to analyze the colocalization of circ-Calm4 and mir-124-3p. The horizontal and vertical axes of the scatter plot are respectively marked with the gray value of each pixel in each channel. Pearson coefficient is shown in the results. The value ranges are from −1 to 1 and the results are close to 1, indicating a stronger correlation.

### Flow Cytometry Assay

PASMCs were transfected with miR-124-3p mimic, miRNA-NC, AMO-124-3p, circ-Calm4 siRNA and *Pdcd6* siRNA in the presence or absence of hypoxia. Cells were collected using trypsin and washed with PBS. Pyroptosis was detected using an Annexin V-FITC/propidium iodide (PI) Kit (Beyotime Biotechnology, Shanghai, China). Cells were stained with annexin V-FITC and PI for 15 minutes according to the manufacturer’s instructions. Pyroptosis was detected with a flow cytometer.

### Immunofluorescence

PASMCs were cultured on sterile glass coverslips in 24-well plates and washed with cold TBST 4 times. Cells were fixed with 4% paraformaldehyde for 15 minutes. Then, the cell membrane was permeabilized with 0.3% Triton X-100 for 1 hour and blocked with normal goat serum for 1 hour at 37 °C. Cells were incubated with an anti-α-SMA antibody (4 µg/mL; BOSTER), an anti-Caspase-1 antibody (10 µg/mL; BOSTER), an anti-Caspase-3 antibody (5.4 µg/mL; Proteintech), an anti-Caspase-9 antibody (4 µg/mL Beyotime Biotechnology, Shanghai, China), anti-Ki-67 antibody (10 µg/mL; BOSTER), anti-Cleaved Caspase-3 (5 µg/mL;Cell Signaling Technology), and an anti-IL-18 antibody (7.6 µg/mL; Proteintech) overnight at 4 °C and were subsequently incubated with FITC-conjugated goat anti-rabbit and Cy3-conjugated goat anti-mouse antibodies for 2 hours at 37 °C. Cells were then washed with TBST, and nuclei were stained with DAPI (Beyotime Biotechnology, Shanghai, China) for 15 minutes at room temperature. ImageJ was used to detect mean gray value, the mean gray value (mean)=integrated density (IntDen)/area, 3 to 5 pictures and 20 to 40 individual cells were measured in different regions of the stained sections. For background correction, we used the default threshold to eliminate the error caused by manually selecting the threshold.

### Hoechst 33342/PI Fluorescence Staining

Pyroptosis was assessed using a Hoechst 33342/PI double fluorescence staining approach (Beyotime Biotechnology, Shanghai, China). PASMCs were cultured in 24-well plates and transfected with miR-124-3p mimic, miRNA-NC, AMO-124-3p, Circ-Calm4 siRNA, and *Pdcd6* siRNA in the presence or absence of hypoxia. Next, cells were stained with 10 μL of Hoechst 33342 solution at 37 °C in the dark for 10 minutes and were then stained with 5 μL of PI at 25 °C in the dark for 15 minutes. The stained cells were observed under a microscope.

### Lactate Dehydrogenase Release Assay

PASMCs were cultured in 96-well plates, and 20 µL of LDH (lactate dehydrogenase) release agent was added to the control group. Supernatants were collected and detected using an LDH assay kit (Beyotime Biotechnology, Shanghai, China). Briefly, 120 μL of cell supernatant was collected and centrifuged at 400×*g* for 5 minutes. Then, 60 μL of LDH test working fluid was added to the samples and incubated at room temperature for 30 minutes. The absorbance was measured at 490 nm in a spectrophotometric microplate reader.

### CCK8 Assay

PASMCs were seeded into 96-well plates, and 10 μL CCK8 kit was added into each well (Beyotime Biotechnology, Shanghai, China). Incubating at 37 °C for 4 hours, the optical density was detected by a spectrophotometer at the wavelength of 450 nm.

### Pull-Down Assay

The pull-down assay was performed by RNA Antisense Purification (RAP) Kit (BersinBioTM, Catalog Bes5103). In vivo, establishing hypoxia-induced PH mice model, dissected pulmonary arterials, and denuded PA vascular endothelium with an iron wire. RNA from denuded PA endothelium vessels were performed pull down assays. In vitro, After 24 hours, ≈2×10^7^ PASMCs were washed in PBS, cells were lysed by Lysis Buffer (BersinBioTM, Catalog Bes5103), and used to perform pull down assays according to the manufacturer’s instructions. The RNA complex was pulled down by incubating the cell lysates with Biotin-labeled circ-calm4 probes (GeneChem Shanghai, China) at 37 °C for 3 hours and streptavidin beads for 30 minutes. The beads were washed 3× with Wash Buffer (BersinBioTM, Catalog Bes5103), and the complexes of RNA were then treated with RNA Elution Buffer (BersinBioTM, Catalog Bes5106) for further purification. The miR-124-3p level was analyzed using quantitative real-time PCR (qRT-PCR).

The primer and probe sequences were as follows:

Circ-Calm4:UGCUCCUGCGG UUUCUGGUAG GAAGC GCC CAUUCCA

NC-circ-Calm4:UGGA AUG GGC GCUU CCUACCAG AAAC CGCAG GAGCA

### RNA Immunoprecipitation Assay

The RNA immunoprecipitation assay was performed by RNA Immunoprecipitation (RIP) Kit (BersinBioTM, Catalog Bes5101). Briefly, 1×10^7^ PASMCs were washed in PBS and lysed with RIP buffer. Then, samples were incubated with containing protein A/G beads (BersinBioTM, Catalog Bes5101) conjugated anti-Ago2 antibodies (5 µg; Proteintech), and immunoglobulin G (5 µg; BersinBioTM, Catalog Bes5101). After extracting RNA, the circ-Calm4 and mir-124-3p level were analyzed by qRT-PCR.

### Absolute Copy Numbers of Per Cell

Absolute copy numbers of circ-Calm4, mir-124-3p, and *Pdcd6* were performed by RT-qPCR in respect to the standard curves. Take the natural log of copy numbers as the Y-coordinate (lgN) and the measured CT value as the X-coordinate, draw the standard curve. The linear equation of the form Y=X+B, B is the y-intercept from the fit linear regression. The copy numbers was then calculated by quantifying the standard substance and by plotting them to serially diluted standard curves. The copies of circ-Calm4 per cell, copies of miR-124-3p per cell, and copies of *Pdcd6* per cell were calculated based on the number of cells.

### Correlations Analysis

Total RNA samples were extracted from lung tissue using TRIZOL reagent (Invitrogen) and for each sample, 500 ng of total RNA was converted to cDNA using a Superscript First-Strand cDNA Synthesis Kit (Invitrogen). To measure the mRNA expression levels of circ-Calm4, miR-124-3p and *Pdcd6*, real-time PCR was used. Threshold cycle (Ct) was determined and relative mRNA and miRNA levels were calculated based on the Ct values. The data were analyzed using the 2^−^^ΔΔCT^ method. The correlation analysis was used by Pearson correlation test. *P* of <0.05 were considered significant. The statistical were analyzed using GraphPad Prism 6.0.

### Data Analysis

Data are presented as the means±SD. All expression values were checked for normal distribution before statistical testing. The 2-tailed Student *t* test was used to compare the data between 2 groups and 1-way ANOVA with Bonferroni post hoc test was used to compare between multiple groups. For comparison between groups with non-normally distributed data, we performed nonparametric analyses such as the Mann-Whitney *U* test for 1 or two groups or Kruskal-Wallis test followed by Dunn post-test for > 2 groups. *P*<0.05 were considered statistically significant. Data were analyzed using GraphPad Prism 6.0.

## Results

### Circ-Calm4 Participates in Pulmonary Arterial Hypertension Though Pyroptosis In Vitro

**Figure 1.**
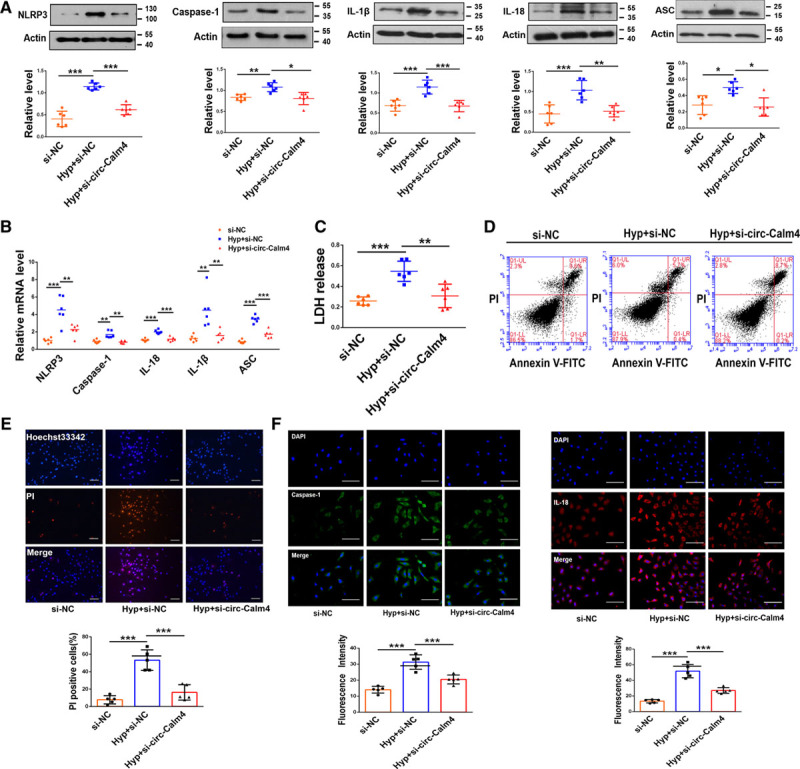
**Circ-Calm4 small-interfering RNA (siRNA) inhibits pyroptosis in pulmonary artery smooth muscle cells (PASMCs).** **A** and **B**, Circ-Calm4 siRNA reversed the increased protein and mRNA levels of Caspase-1, NLRP3 (nucleotide-binding oligomerization segment-like receptor family 3), ASC (apoptosis-associated speck-like protein containing a caspase recruitment segment), IL-18 (interleukin-18), and IL-1β (interleukin-1β) induced by hypoxia in PASMCs (n=6). **C**, Circ-Calm4 siRNA reversed the increased LDH (lactate dehydrogenase) activity in PASMCs subjected to hypoxia for 24 h. LDH release was evaluated with an LDH release kit (n=6). **D**, Knockdown of circ-Calm4 decreased the number of pyroptotic cells in PASMCs exposed to hypoxia. Cells were detected with annexin V-FITC/propidium iodide (PI) double staining using quantitative fluorescence-activated cell sorting (FACS) analysis. **E**, Knockdown of circ-Calm4 by siRNA reduced the positive PI staining induced by hypoxia in PASMCs. Scale bars=100 µm. Images of fluorescence staining with PI (red) and Hoechst 33342 (blue). **F**, Circ-calm4 siRNA attenuated the fluorescence staining of Caspase-1 and IL-18 induced by hypoxia in PASMCs. Scale bars=100 µm. Cells were stained for Caspase-1 (green) and IL-18 (red), and DAPI (blue) was used for nuclear staining. Each datapoint in the figure represents a unique biological replicate. The data are presented as the means±SD. Statistical analysis was performed with 1-way ANOVA followed by Bonferroni correction. The graph A-ASC and Caspase-1 were analyzed by the Kruskal-Wallis test followed by Dunn post-test. *P<0.05, **P<0.01, ***P<0.001.

To determine the expression of circ-Calm4 in PH, we investigated the expression of the circ-Calm4 using quantitative PCR. The results showed that circ-Calm4 was markedly upregulated in the lungs and (mouse) PASMCs under hypoxia (Figure IA and IB in the Data Supplement), which was consistent with the finding using the RNA FISH (Figure IC in the Data Supplement). Therefore, we examined the subcellular localization of circ-Calm4, we found that circ-Calm4 was detected in both the cytoplasm and nucleus of PASMCs (Figure ID in the Data Supplement). Moreover, we isolated cytoplasmic and nuclear RNA and observed that circ-Calm4 localized both in the cytosol and the nucleus and was markedly upregulated in PASMCs under hypoxia by quantitative PCR analysis (Figure IE in the Data Supplement). To evaluate the possible release of circ-Calm4, we analyzed circulating circ-Calm4 levels in mice and medium from PASMCs. Results showed that circ-Calm4 was increased compared with normoxia (Figure IF and IG in the Data Supplement). To clarify whether circ-Calm4 plays a role in PASMC pyroptosis, we transfected circ-Calm4 siRNA into PASMCs to silence endogenous circ-Calm4. The transfection efficiency was verified by a significant decrease in endogenous circ-Calm4 after siRNA transfection, and we found that si-circ-Calm4-2 had the most obvious effects in a time-dependent manner exposed to hypoxia (Figure IH in the Data Supplement). Nevertheless, cells transduced with si-circ-Calm4-2 did not alter the expression of lincalm4 in PASMCs exposed to hypoxia (Figure II in the Data Supplement). PASMCs transduced with circ-Calm4 overexpression vector following real-time RT-PCR to analysis lincalm4 expression. The results showed that circ-Calm4 overexpression did not alter the expression of lincalm4 (Figure IJ and IK in the Data Supplement). The pyroptosis-related proteins NLRP3, Caspase-1, IL-1β, IL-18, and ASC were downregulated in hypoxia-exposed PASMCs by silencing circ-Calm4 (Figure 1A). We also transfected circ-Calm4 siRNA into PASMCs to silence endogenous circ-Calm4 to identify the effect of knockdown circ-Calm4 on activated Caspase 1. The results showed that the protein expression levels of activated Caspase 1 were decreased by the circ-Calm4 siRNA in PASMCs exposed to hypoxia (Figure IL in the Data Supplement). Similarly, hypoxia-induced increases in the mRNA expression levels of *Nlrp3, Caspase-1, Il-1β, Il-18*, and *Asc* were alleviated by circ-Calm4 siRNA (Figure 1B). As shown in Figure 1C, the LDH release assay results indicated that circ-Calm4 siRNA attenuated the increased LDH activity in PASMCs exposed to hypoxia. In addition, the Annexin V-FITC/PI double staining results showed that circ-Calm4 siRNA abrogated pyroptotic cell death induced by hypoxia (Figure 1D). The increased positive PI staining in hypoxia-exposed PASMCs cells decreased distinctly after transfection of circ-Calm4 siRNA (Figure 1E). Immunostaining for Caspase-1 and IL-18 showed that circ-Calm4 siRNA diminished the increase in fluorescence activity induced by hypoxia (Figure 1F). To further examine the function of circ-Calm4 in PASMC pyroptosis, we transfected circ-Calm4 plasmid for overexpression. The pyroptosis-related proteins and mRNA levels were upregulated in PASMCs by circ-Calm4-plasmid (Figure IM and IN in the Data Supplement). As linear and circRNA isoforms may have independent functions in both normal and pathological conditions.^33^ We performed quantitative PCR to detect the expression of lincalm4 in different tissues, we found that lincalm4 was markedly increased in the lungs (Figure IIA in the Data Supplement). Furthermore, we determined that lincalm4 was expressed in macrophage, PASMCs, and PAECs, with the highest expression in PASMCs under hypoxia (Figure IIB in the Data Supplement). To reveal the action of lincalm4 in PASMC pyroptosis, we transfected lincalm4 siRNA into PASMCs to silence endogenous lincalm4, and we found that si-lincalm4-3 had the most obvious effects (Figure IIC in the Data Supplement). The protein and mRNA expression levels of pyroptosis-related molecular markers were unchanged by the lincalm4 siRNA in PASMCs exposed to hypoxia (Figure IID and IIE in the Data Supplement).

### Circ-Calm4 Regulates Pulmonary Arterial Hypertension Though Pyroptosis In Vivo

**Figure 2.**
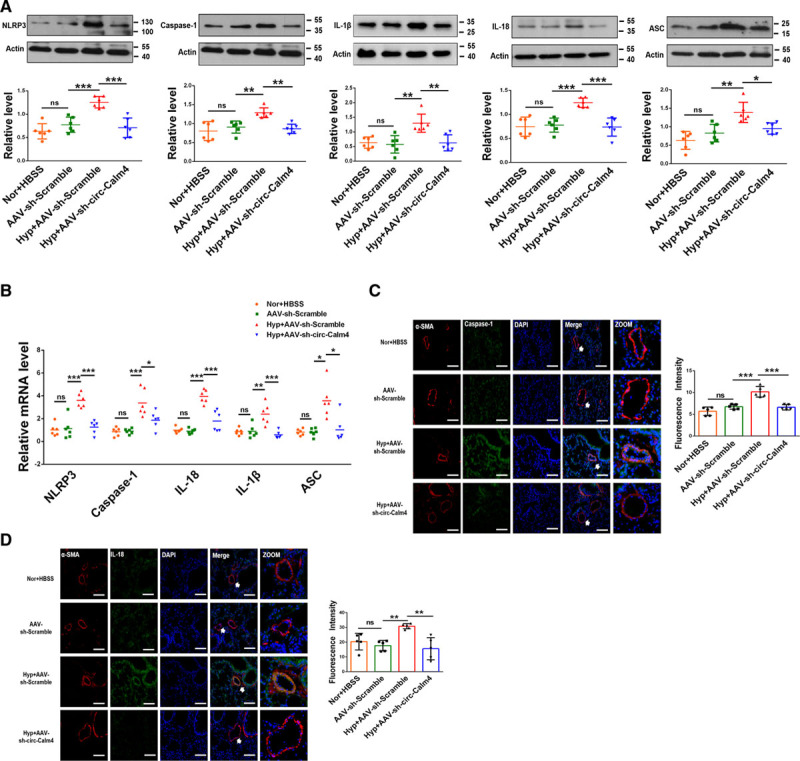
**Circ-Calm4 inhibits pyroptosis in a mouse model of hypoxia-induced pulmonary hypertension (PH).** **A** and **B**, Knockdown of circ-Calm4 by sh-circ-Calm4 countered the hypoxia-induced upregulation of Caspase-1, NLRP3 (nucleotide-binding oligomerization segment-like receptor family 3), ASC (apoptosis-associated speck-like protein containing a caspase recruitment segment ),IL-18 (interleukin-18), and IL-1β (interleukin-1β) at the protein and mRNA levels (n=6; in Western blot, the image of NLPR3 and Caspase-1 used the same actin). **C** and **D**, Knockdown of circ-Calm4 by sh-circ-Calm4 reversed the hypoxia-induced upregulation of Caspase-1 and IL-18 in mouse lung tissues. Scale bars=100 µm. Lung sections stained with Caspase-1 (green) and IL-18 (green), pulmonary smooth muscle stained with α-SMA (red), and DAPI for nuclear staining. Each datapoint in the figure represents a unique biological replicate. The data are presented as the means±SD. Statistical analysis was performed with 1-way ANOVA followed by Bonferroni correction. The graph B-ASC was analyzed by the Kruskal-Wallis test followed by Dunn post-test. *P<0.05, **P<0.01, ***P<0.001.

We further aimed to understand the function of circ-Calm4 in the PH model. Mice were allowed to inhale Hanks’ Balanced Salt Solution, AAV9 viral particles carrying sh-circ-Calm4 or the scrambled fragment via dropwise intranasal instillation for 10 days before establishment of the hypoxia-induced PH model. The abundance of circ-calm4 was markedly decreased by sh-circ-Calm4 in mouse lung tissues (Figure IIIA in the Data Supplement). Both the right ventricular systemic pressure and the right ventricle-to-left ventricle plus septum (RV/LV+S) ratio were lower in circ-Calm4-silenced hypoxic mice than in mice treated with the scrambled fragment (Figure IIIB and IIIC in the Data Supplement). Echocardiographic analysis showed that the pulmonary artery acceleration time and pulmonary arterial velocity time integral were greater in circ-Calm4-silenced hypoxic mice than in scramble-treated hypoxic mice (Figure IIID in the Data Supplement). Consistent with these results, the protein and mRNA expression levels of NLRP3, Caspase-1, IL-1β, IL-18, and ASC were significantly lower in sh-circ-Calm4 mouse lung tissues than in scramble-treated mouse lung tissues after 21 days of hypoxia exposure (Figure 2A and 2B). Immunofluorescence staining for Caspase-1 and IL-18 revealed that the fluorescence activity in sh-circ-Calm4 mouse lung tissues was decreased compared with that in scramble-treated mouse lung tissues (Figure 2C and 2D). We also examined the function of circ-Calm4 in cell proliferation and cell apoptosis through immunofluorescence staining of Ki67 and Caspase-3. The data showed that circ-Calm4 knockdown by sh-circ-Calm4 prevented increases in Ki67 expression in hypoxia-treated mice (Figure IIIE in the Data Supplement). HIF1-α was used as a positive control of hypoxia marker. Studies have confirmed that the inhibition of apoptosis of PASMCs is the main pathological change in PH, which results in pulmonary vascular remodeling. Our results showed silencing circ-Calm4 increased the expression of Caspase-3 compared with the negative control group (Figure IIIF in the Data Supplement). We also used IF images to investigate the activated Caspase 3 mechanism in vivo. Our results showed silencing circ-Calm4 increased the expression of cleaved Caspase-3 compared with that in control vector mouse lung tissues (Figure IIIG in the Data Supplement).

### Circ-Calm4 Acts As a Competing Endogenous RNA for miR-124-3p in PASMCs

**Figure 3.**
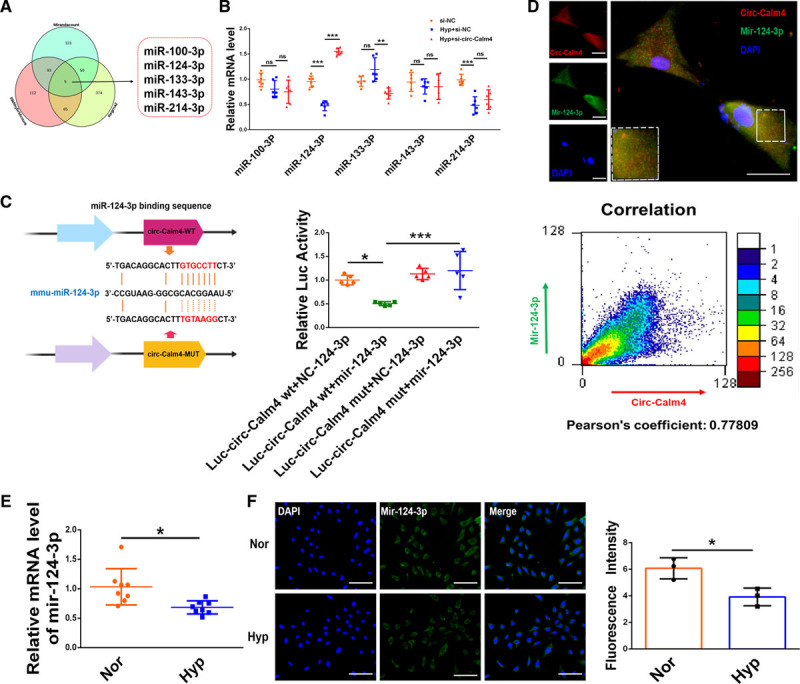
**Circ-calm4 is a mediator that regulates miR-124-3p action.** **A**, The target microRNAs (miRNAs) of circ-Calm4, as predicted by the miRanda Count, RNA hybrid Count and RegRNA 2.0 databases. **B**, Knockdown of circ-Calm4 by small-interfering RNA increased the miR-124-3p mRNA level in pulmonary arterial smooth muscle cells (PASMCs) exposed to hypoxia for 24 h (n=6). **C**, HEK293 cells were cotransfected with a luciferase reporter construct carrying wild-type (WT) or mutant (Mut) circ-Calm4 and miR-124-3p or miR-124-3p-NC. Luciferase activities were measured via a dual luciferase assay (n=5). **D**, Colocalization of circ-Calm4 and miR-124-3p in PASMCs. Scale bars=100 µm. MiR-124-3p probes were labeled with FAM (green), whereas circ-Calm4 probes were labeled with Cy3 (red). Nuclei were stained with DAPI (blue). Pearson coefficient is 0.77809, indicating a correlation. **E**, Downregulation of miR-124-3p in PASMCs exposed to hypoxia for 24 h relative to miR-124-3p expression in nontreated cells (n=8). **F**, Relative fluorescence of miR-124-3p in PASMCs exposed to hypoxia. Scale bars=100 µm. miR-124-3p probes were labeled with FAM (green). Nuclei were stained with DAPI (blue). Each datapoint in the figure represents a unique biological replicate. The data are presented as the means±SD. Statistical analysis was performed with 1-way ANOVA followed by Bonferroni correction and Student *t* test for 2 means. *P<0.05, **P<0.01, ***P<0.001.

As circ-Calm4 is abundant in the cytoplasm, we investigated whether it regulated PH via the ceRNA (competing endogenous RNA) mechanism. We performed bioinformatic analysis using the miRanda Count database, RNAhybrid Count database, and RegRNA 2.0 database to predict the miRNAs with the potential target sequence to bind to circ-Calm4 (Figure 3A). Via analysis of those databases, miR-100-3p, miR-124-3p, miR-133-3p, miR-143-3p, and miR-214-3p were selected for further study. Among these miRNAs, the level of only miR-124-3p was increased after silencing circ-Calm4 in hypoxic PASMCs (Figure 3B). To get rid of the effect of mir-124-3p changes induced by linear transcript, we transfected lincalm4 siRNA into PASMCs to silence endogenous lincalm4 following real-time RT-PCR to analysis mir-124-3p expression. The results showed that lincalm4 siRNA did not alter the expression of mir-124-3p (Figure IVA in the Data Supplement). Meanwhile, we constructed the lincalm4 plasmid and transfected into PASMCs to overexpress lincalm4 following real-time RT-PCR to analysis of mir-124-3p expression. The results showed that lincalm4 overexpression did not alter the expression of mir-124-3p (Figure IVB and IVC in the Data Supplement). Next, qRT-PCR assays displayed that overexpression of circ-Calm4 resulted in downregulation of mir-124-3p in hypoxic PASMCs (Figure IVD in the Data Supplement). We also identified the effect of circ-Calm4 siRNA and lincalm4 siRNA on mir-124-3p mRNA level. The qPCR results showed that the expression levels of mir-124-3p was reduced in hypoxia-treated PASMCs, and the effects were effectively reversed by knockdown circ-Calm4 in hypoxia-treated PASMCs. Next, the levels of mir-124-3p were detected in PASMCs co-transfected with circ-Calm4 siRNA and lincalm4 siRNA under hypoxic condition, the results showed the expression levels of mir-124-3p did not alter by cotransfection with circ-Calm4 siRNA and lincalm4 siRNA compared with those transfected with circ-Calm4 siRNA in hypoxia-treated PASMCs (Figure IVE in the Data Supplement). However, neither transfecting miR-124-3p mimic nor AMO-124-3p altered the expression of circ-Calm4 or lincalm4 in hypoxic PASMCs (Figure IVF through IVH in the Data Supplement). We also found that mir-124-3p localized both in the cytosol and the nucleus by quantitative PCR analysis. Nevertheless, miR-124-3p is only downregulated in the cytoplasm of PASMCs under hypoxia (Figure IVI in the Data Supplement). Thus, we focused our further analysis on circ-Calm4 and miR-124-3p. Sequence analysis showed that there are 2 potential binding sites for miR-124-3p in circ-Calm4, potential seed region(red) of miR-124-3p were found completely complementary with the circ-Calm4 sequence 511 to 529 (Figure IVJ in the Data Supplement). We also used Website of the MEME to describe the potential of base pairing, we used the position *P* to describe the potential of base pairing, the computational motif analysis of circ-calm4 were *P*=1.69×10^−9^ and *P*=3.19×10^−17^ of the 2 miR-124-3p targets sites. We also describe the combined match *P* of the base pairing and the result is 6.42×10^−29^. The statistical significance of the motif is the e-value of MEME and the result is 1.9×10^−3^. The MEME predictions analyses showed the exact extent of hybridization between circ-calm4 and miR-124-3p (Figure IVK in the Data Supplement). The luciferase assay results confirmed that miR-124-3p suppressed the luciferase activity of the circ-Calm4 3′-UTR-wt construct but not the circ-Calm4 3′-UTR-mut construct (Figure 3C). The results of FISH experiments further showed the colocalization of circ-Calm4 and miR-124-3p (Figure 3D). To validate the direct binding between circ-Calm4 and miR-124-3p, we used pull-down assay to pull down endogenous miRNAs associated with circ-Calm4. We established hypoxia-induced PH mice model, dissected pulmonary arterials, and denuded PA vascular endothelium with an iron wire. By using denuded PA endothelium vessels, we performed pull down assays to endogenous circ-Calm4 and miR-124-3p microRNA binding in vivo. Cell lysate from denuded PA vascular endothelium were labeled with biotin-labeled circ-Calm4-specific probe and negative control probe, respectively. RNA complex were pulled down, and miR-124-3p expression was analyzed using qRT-PCR. The results showed that miR-124-3p was significantly enriched in RNAs retrieved from bio-circ-Calm4 compared with that from bio-NC-circ-Calm4. In addition, we also performed pull down endogenous miRNAs associated with circ-Calm4 in hypoxia-exposed PASMCs. Biotin-labeled circ-Calm4-specific probe and negative control probe were used to pulled down the RNA complex, and qRT-PCR to analyze miR-124-3p expression. The results showed that miR-124-3p was significantly enriched in bio-circ-Calm4 compared with that in bio-NC-circ-Calm4. Our results in vivo and in vitro indicated that the specific interaction between circ-Calm4 and miR-124-3p is exist (Figure IVL in the Data Supplement). We then performed an assay for AGO2 in PASMCs overexpressing miR-124-3p and circ-Calm4, respectively, endogenous circ-Calm4 and mir-124-3p were significantly enriched in AGO2 antibodies compared with control IgG antibodies. But, at endogenous level, circ-Calm4 is in fact not detectable in AGO2 complexes compared with IgG controls, only after experimental miR-124-3p overexpression, an AGO2:circCalm4 interaction can be seen. Conversely, only after circCalm4 overexpression, an AGO2: miR-124-3p interaction is detectable. A stable AGO2 interaction is normally not seen, unless enforced by artificial overexpression (Figure IVM in the Data Supplement). Absolute copies per cell of circ-Calm4, mir-124-3p, and *Pdcd6* were performed by quantitative PCR analysis in respect to their standard curves (Figure IVN in the Data Supplement). Moreover, the qPCR and FISH experiment results showed that the level of miR-124-3p was markedly downregulated upon exposure to hypoxia (Figure 3E and 3F; Figure IVO in the Data Supplement).

### The Role of miR-124-3p in PASMC Pyroptosis

**Figure 4.**
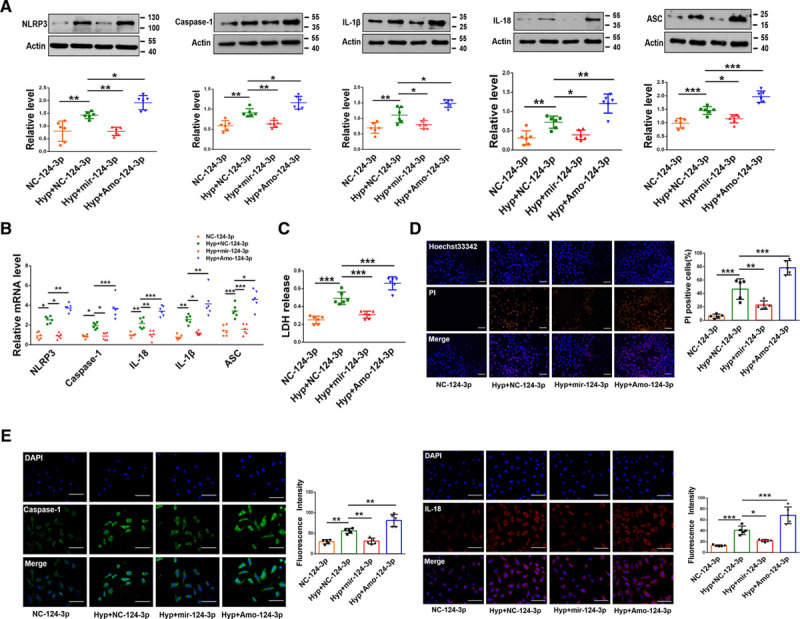
**MiR-124-3p suppresses pyroptosis in pulmonary arterial smooth muscle cells (PASMCs).** **A** and **B**, In PASMCs exposed to hypoxia, transfection of the miR-124-3p mimic decreased the protein and mRNA levels of Caspase-1, NLRP3 (nucleotide-binding oligomerization segment-like receptor family 3), ASC (apoptosis-associated speck-like protein containing a caspase recruitment segment), IL-18 (interleukin-18), and IL-1β (interleukin-1β); in contrast, AMO-124-3p increased these protein and mRNA levels (n=6; in Western blot, the image of IL-1β and IL-18 used the same actin.). **C–E**, Transfection of the miR-124-3p mimic ameliorated pyroptosis-related phenotypes upon hypoxia treatment, whereas AMO-124-3p exacerbated these phenotypes. LDH (lactate dehydrogenase) activity (n=6; **C**). Propidium iodide (PI)-positive stained cells. Scale bars=100 µm. Images of fluorescence staining with PI (red) and Hoechst 33342 (blue) (**D**). Fluorescence staining for Caspase-1 and IL-18. Cells were stained for Caspase-1 (green) and IL-18 (red), and DAPI (blue) was used for nuclear staining (**E**). Scale bars=100 µm. Each datapoint in the figure represents a unique biological replicate. The data are presented as the means±SD. Statistical analysis was performed with 1-way ANOVA followed by Bonferroni correction. *P<0.05, **P<0.01, ***P<0.001.

We further determined the role of miR-124-3p in pyroptosis by treating PASMCs with a miR-124-3p mimic and AMO-124-3p, an inhibitor of miR-124-3p. The protein and mRNA expression levels of NLRP3, Caspase-1, IL-1β, IL-18, and ASC were decreased by the miR-124-3p mimic in PASMCs exposed to hypoxia, and these effects were significantly enhanced by AMO-124-3p (Figure 4A and 4B; Figure VA and VB in the Data Supplement). Moreover, LDH activity and positive PI staining in cells were abrogated by the miR-124-3p mimic in response to hypoxic stimulation, and AMO-124-3p enhanced both phenotypes (Figure 4C and 4D). The fluorescence activity of Caspase-1 and Il-18 was minimized after transfection with the miR-124-3p mimic under hypoxia exposure, and this decrease was reversed by AMO-124-3p (Figure 4E).

### Circ-Calm4 Promotes Pyroptosis via Acting As a Molecular Sponge to Adsorb miR-124-3p in PASMCs

**Figure 5.**
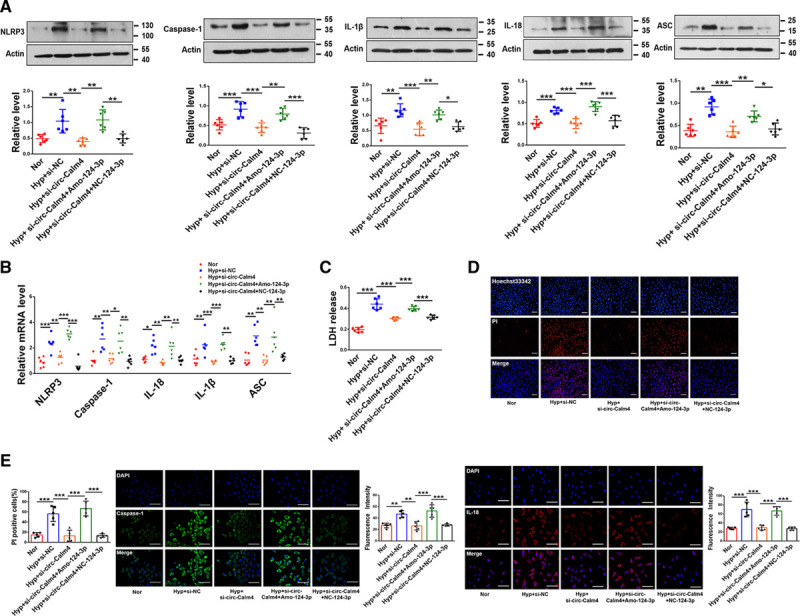
**Circ-Calm4 sponges miR-124-3p to mediate pulmonary arterial smooth muscle cell (PASMC) pyroptosis.** **A** and **B**, Knockdown of endogenous miR-124-3p by AMO-124-3p abrogated the antipyroptotic effects of Circ-calm4 silencing by Circ-calm4-small-interfering RNA (siRNA) upon hypoxia exposure in PASMCs, as indicated by the protein and mRNA levels of Caspase-1, NLRP3 (nucleotide-binding oligomerization segment-like receptor family 3), ASC (apoptosis-associated speck-like protein containing a caspase recruitment segment), IL-18 (interleukin-18), and IL-1β (interleukin-1β; n=6). **C**, Cotransfection with circ-Calm4 siRNA and AMO-124-3p abrogated the antipyroptotic effects on PASMCs upon hypoxia exposure, as indicated by LDH (lactate dehydrogenase) activity (n=6). **D**, Knockdown of circ-Calm4 by siRNA reduced the positive propidium iodide (PI) staining induced by hypoxia in cells, whereas AMO-124-3p reversed the decrease in PI-positive PASMCs. Scale bars=100 µm. Images of fluorescence staining with PI (red) and Hoechst 33342 (blue). **E**, Circ-Calm4 knockdown blocked the fluorescence intensity of Caspase-1 and IL-18 under hypoxia exposure, and this decrease was abrogated after transfection of AMO-124-3p. Scale bars=100 µm. Cells were stained for Caspase-1 (green) and IL-18 (red), and DAPI (blue) was used for nuclear staining. Each datapoint in the figure represents a unique biological replicate. The data are presented as the means±SD. Statistical analysis was performed with 1-way ANOVA followed by Bonferroni correction. *P<0.05, **P<0.01, ***P<0.001.

The reverse experiments were performed to evaluate the regulation of pyroptosis by circ-Calm4 via miR-124-3p. The Western blot and qPCR results showed that the expression levels of NLRP3, Caspase-1, IL-1β, IL-18, and ASC were reduced by circ-Calm4 silencing in hypoxia-treated PASMCs, while this downregulation was prevented by cotransfection with AMO-124-3p (Figure 5A and 5B). Cotransfection of Amo-124-3p reversed the protective effects of circ-Calm4 siRNA on hypoxia-induced pyroptosis in PASMCs, as exhibited by the LDH activity and positive PI staining in cells (Figure 5C and 5D). Similarly, circ-Calm4 siRNA suppressed the hypoxia-induced increase in immunofluorescence staining; however, in the presence of AMO-124-3p, circ-Calm4 siRNA lost its ability to reverse the hypoxia-induced upregulation of Caspase-1 and IL-18 (Figure 5E).

### PDCD6 Is a Mediator of miR-124-3p Action

**Figure 6.**
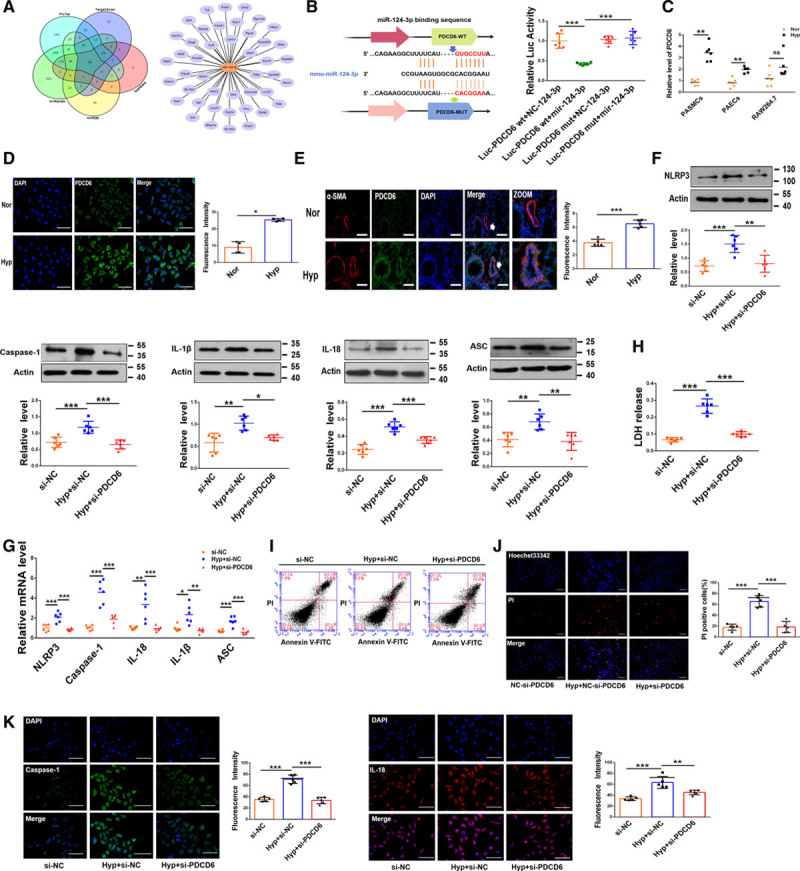
**PDCD6 (programmed cell death protein 6) is a downstream target gene of miR-124-3p.** **A**, The target genes of miR-124-3p, as predicted by the TargetScan, PicTar, StarBase, miRDB and miRanda databases. **B**, HEK293 cells were cotransfected with a luciferase reporter construct carrying wild-type (WT) or Mut *Pdcd6* and miR-124-3p or miR-124-3p-NC. Luciferase activities were measured via a dual luciferase assay (n=6). **C**, The expression of *Pdcd6* in different cells (n=6). **D**, Upregulation of *Pdcd6* in hypoxic pulmonary arterial smooth muscle cells (PASMCs) relative to normal cells. Scale bars=100 µm. Cells were stained for PDCD6 (green), and DAPI (blue) was used for nuclear staining. **E**, Upregulation of PDCD6 in hypoxic mice. Scale bars=100 µm. Lung sections were stained for PDCD6 (green), pulmonary smooth muscle was stained for α-SMA (red), and DAPI was used for nuclear staining. **F** and **G**, Si-*Pdcd6* countered the hypoxia-induced upregulation of Caspase-1, NLRP3 (nucleotide-binding oligomerization segment-like receptor family 3), ASC (apoptosis-associated speck-like protein containing a caspase recruitment segment), IL-18 (interleukin-18), and IL-1β (interleukin-1β) expression at the protein and mRNA levels in PASMCs (n=6). **H**, Si-PDCD6 reversed the hypoxia-induced upregulation of LDH activity in PASMCs (n=6). **I**, Knockdown of *Pdcd6* decreased the number of pyroptotic PASMCs upon hypoxia exposure. Cells were analyzed by annexin V-FITC/propidium iodide (PI) double staining using quantitative FACS analysis. **J**, *Pdcd6* silencing reduced the positive PI staining induced by hypoxia in PASMCs. Scale bars=100 µm. Images of fluorescence staining with PI (red) and Hoechst 33342 (blue). **K**, Si-PDCD6 attenuated the fluorescence staining for Caspase-1 and IL-18 induced by hypoxia in PASMCs. Scale bars=100 µm. Cells were stained for Caspase-1 (green) and IL-18 (red), and DAPI (blue) was used for nuclear staining. Each datapoint in the figure represents a unique biological replicate. The data are presented as the means±SD. Statistical analysis was performed with 1-way ANOVA followed by Bonferroni correction and Student *t* test for 2 means. The graph D was analyzed by the Mann-Whitney *U* test and the graph F-IL-1β, the graph G-*Ii-1β* was analyzed by the Kruskal-Wallis test followed by Dunn post-test. *P<0.05, **P<0.01, ***P<0.001.

To further investigate the target genes of miR-124-3p, we performed an integrated bioinformatic prediction analysis with the PicTar, TargetScan, miRanda, miRDB, and StarBase databases. Forty-six genes were predicted to be potential targets of miR-124-3p (Figure 6A). Intriguingly, miR-124-3p regulates *Pdcd6* directly.^34^ In agreement with the previous studies, the luciferase assay results revealed that miR-124-3p significantly inhibited luciferase activity in PASMCs transfected with *Pdcd6* 3′-UTR-wt but not in those transfected with *Pdcd6* 3′-UTR-mut (Figure 6B). In addition, we transfected *Pdcd6* siRNA into PASMCs to silence *Pdcd6* and si- *Pdcd6*-2 had the most obvious effects (Figure VIA in the Data Supplement). *Pdcd6* mRNA levels were significantly increased in hypoxia-exposed PASMCs (Figure 6C), and immunofluorescence staining for PDCD6 was markedly increased in hypoxia-exposed PASMCs (Figure 6D). Consistent with the results of the in vitro experiments described above, the level of PDCD6 was also increased in hypoxia-treated mice (Figure 6E). To clarify whether *Pdcd6* plays a role in hypoxia-induced pyroptosis, PASMCs were transfected with *Pdcd6* siRNA. *Pdcd6* siRNA transfection decreased the protein and mRNA expression levels of NLRP3, Caspase-1, IL-1β, IL-18, and ASC upon hypoxic stimulation (Figure 6F and 6G). We further observed that knockdown of *Pdcd6* decreased the level of LDH activity induced by hypoxia (Figure 6H). Under hypoxia exposure, pyroptotic cell death was reversed by *Pdcd6* siRNA (Figure 6I). Consistent with this result, *Pdcd6* siRNA diminished the hypoxia-induced positive PI staining in cells (Figure 6J). Furthermore, transfection of *Pdcd6* siRNA decreased the levels of Caspase-1 and IL-18 in PASMCs exposed to hypoxia (Figure 6K). We further investigated the potential effects of *Pdcd6* in hypoxia-induced proliferation in PASMCs with CCK8. The results show that *Pdcd6* siRNA diminished the hypoxia-induced proliferation in PASMCs (Figure VIB in the Data Supplement).

### Circ-Calm4 Regulates Pulmonary Arterial Hypertension via the miR-124-3p/PDCD6 Axis

**Figure 7.**
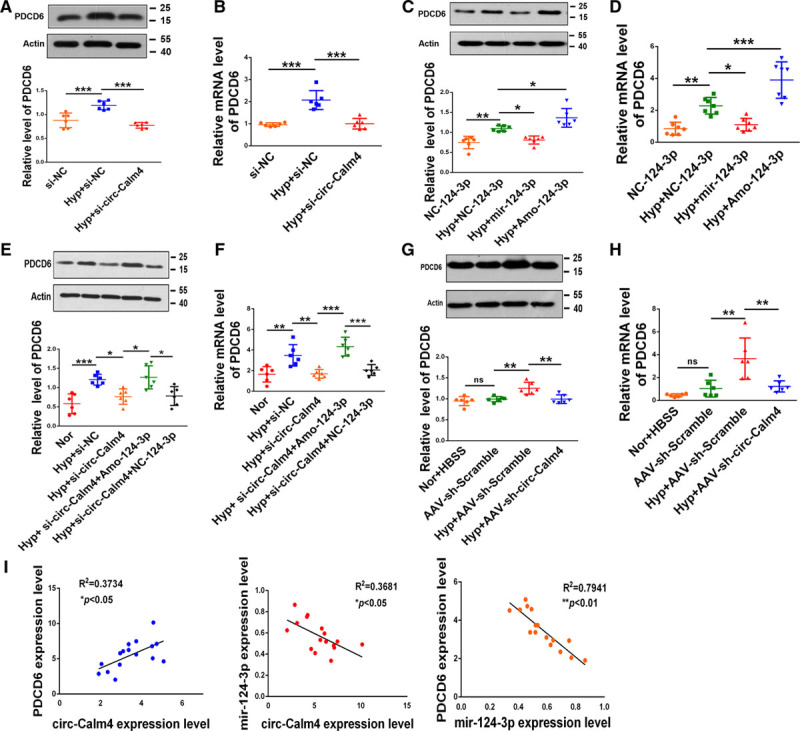
**Circ-Calm4 small-interfering RNA inhibits pyroptosis in pulmonary arterial smooth muscle cells (PASMCs) through the miR-124-3p/PDCD6 (programmed cell death protein 6) pathway.** **A** and **B**, Knockdown of endogenous circ-Calm4 abrogated upregulation of PDCD6 protein and *Pdcd6* mRNA expression induced by hypoxia (n=6). **C** and **D**, MiR-124-3p reversed but AMO-124-3p enhanced the upregulation of PDCD6 protein and *Pdcd6* mRNA expression induced by hypoxia (n=6). **E** and **F**, In PASMCs, knockdown of circ-Calm4 reduced PDCD6 protein and *Pdcd6* mRNA expression induced by hypoxia, whereas AMO-124-3p reversed the downregulation of PDCD6 protein and *Pdcd6* mRNA expression (n=6). **G** and **H**, Knockdown of circ-Calm4 by sh-circ-Calm4 countered the hypoxia-induced upregulation of PDCD6 at the protein and mRNA levels (n=6). **I**, Circ-Calm4 expression was positively correlated with *Pdcd6* mRNA expression in hypoxic mice. In hypoxic mice, circ-Calm4 expression was negatively correlated with miR-124-3p expression, and *Pdcd6* mRNA expression was negatively correlated with miR-124-3p expression. Each datapoint in the figure represents a unique biological replicate. The data are presented as the means±SD. Statistical analysis was performed with 1-way ANOVA followed by Bonferroni correction. *P<0.05, **P<0.01, ***P<0.001.

To validate the role of the circ-Calm4/miR-124-3p/*Pdcd6* axis in hypoxia-induced pyroptosis, we examined the link between circ-Calm4 and miR-124-3p as well as the downstream mediator *Pdcd6*. Transfection of circ-Calm4 siRNA attenuated the protein and mRNA expression of PDCD6 induced by hypoxia in PASMCs (Figure 7A and 7B). We then found that transfection of the miR-124-3p mimic attenuated the PDCD6 level after hypoxia exposure. In contrast, AMO-124-3p enhanced *Pdcd6* expression at both the mRNA and protein levels under hypoxia exposure (Figure 7C and 7D). Moreover, knockdown of circ-Calm4 decreased the protein and mRNA expression levels of PDCD6, while these levels were markedly rescued by the miR-124-3p inhibitor in cultured PASMCs exposed to hypoxia (Figure 7E and 7F). Consistent with the in vitro data, knockdown of circ-Calm4 lessened the upregulation of PDCD6 protein and mRNA expression in anoxic mice (Figure 7G and 7H). As indicated by correlation analysis, the expression level of circ-Calm4 was significantly correlated with that of *Pdcd6* mRNA and circ-Calm4 was positively correlated with lincalm4 (Figure VII in the Data Supplement). In contrast, circ-Calm4 expression was negatively correlated with miR-124-3p expression. Furthermore, the expression levels of miR-124-3p were negatively correlated with those of *Pdcd6* mRNA (Figure 7I).

## Discussion

**Figure 8.**
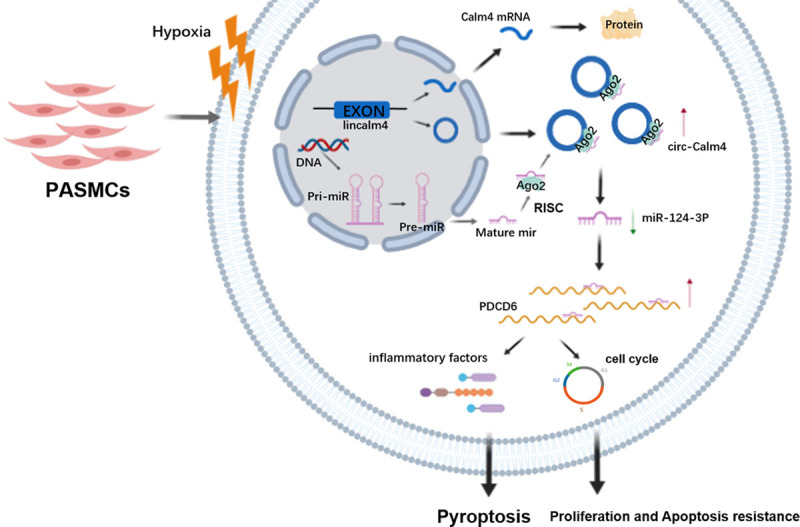
**A schematic diagram to illustrate the hypothetical model Circ-Calm4, as a competitive endogenous RNA, adsorbed miR-124-3p via a circular RNA sponging mechanism and abolished the suppression of *Pdcd6* (programmed cell death protein 6) by miR-124-3p, leading to pyroptosis, proliferation, and apoptosis resistance. PASMC indicates pulmonary arterial smooth muscle cell.**

Previous studies suggested that pyroptosis is associated with the pathogenesis of PH.^35^ CircRNAs with diverse functions may regulate signaling pathways and targets related to pyroptosis in PASMCs. Our study demonstrates this hypothesis from the following 3 perspectives. First, we identified that circ-Calm4 was markedly upregulated in hypoxia-induced PH in vivo and in vitro. Second, we proved that pyroptosis induced by hypoxia was mediated by circ-Calm4. Third, we elucidated that circ-Calm4, as a ceRNA, adsorbed miR-124-3p via a circRNA sponging mechanism and abolished the suppression of *Pdcd6* by miR-124-3p, leading to pyroptosis. The findings revealed a new signaling pathway of pyroptosis mediated by circ-Calm4 in hypoxic PH (Figure 8).

Pyroptosis, another form of programmed cell death, is involved in many diseases, such as infectious diseases, nervous system diseases, and atherosclerotic diseases.^36^ In addition, the levels of activated Caspase-1 and IL-18 products are reported to be increased in the model of hypoxic PH, suggesting that pyroptosis may occur in PH.^35^ Our study proved that hypoxia induces increases in the protein and mRNA expression levels of Caspase-1, NLRP3, ASC, IL-18, and IL-1β. In addition, PI staining and LDH activity increased under hypoxia exposure. Therefore, our results provide solid evidence that pyroptosis can occur in hypoxic PH.

Circ-Calm4 is a newly identified circRNA that contains the exon sequence spanning positions 3837757 to 3838671 of the *Calm4* gene. A recent study found that

circ-Calm4 might act as a ceRNA to regulate the expression of *Myo10* (Myosin 10) via sponging miR-337-3p to promotes PASMC proliferation.^29^ But, the role and mechanism of circ-Calm4 in pyroptosis have not yet been fully studied. The present study showed that circ-Calm4 was markedly upregulated in hypoxia-induced PH in vivo and in vitro. To investigate the role of circ-Calm4 in pyroptosis, we assessed pyroptosis phenotypes via adenovirus-mediated knockdown or transfection of circ-Calm4 siRNA. Silencing circ-Calm4 mitigated the upregulation of pyroptosis induced by hypoxia, suggesting that PASMC pyroptosis induced by hypoxia is mediated by circ-Calm4.

Theoretically, *Calm4* genes can encode lincalm4 mRNA and lincalm4 proteins besides coding circ-Calm4. It had been reported that back-splicing of single exons of circRNA may affect the splicing of linear RNAs.^37^ CircRNAs may regulate the activity of the linear mRNAs by binding miRNAs.^38^ In our results showed that neither overexpression nor knockdown of circ-Calm4 alter the expression of lincalm4 but affected the expression of miR-124-3p in PASMCs. We further proved that miR-124-3p did not involve in regulation of the expression of lincalm4 in hypoxic PASMCs, suggesting that the regulating mechanisms of lincalm4 expression in hypoxic PASMCs are irrelevant to circ-Calm4 and miR-124-3p. However, in the absence of an CALM4 antibody, we did not know for sure whether circ-Calm4 was potentially encoding and expressing the CALM4 protein; or that miR-124-3p repressed CALM4 protein expression, although it did not cleave *Calm4* transcripts. As linear and circRNAs are generated from the same RNA precursor, it is necessary to consider the function of lincalm4 in hypoxia induced PASMCs pyroptosis. Our results showed that circ-Calm4 and lincalm4 are present in PASMCs, si-circ-Calm4 did not affect the expression of lincalm4 mRNA. More importantly, silencing lincalm4 mRNA to silence endogenous *Calm4* (Figure IIC through IIE in the Data Supplement) does not affect hypoxia induced PASMCs pyroptosis, suggesting that lincalm4 is not involved in hypoxia induced PASMCs pyroptosis.

The function of ceRNA suggests a mechanism whereby RNA molecules affect target RNAs by competitively binding to regions of sequence complementarity.^39^ Indeed, numerous circRNAs have been demonstrated to regulate the progression of various diseases through the ceRNA mechanism.^40–42^ In the present study, we used bioinformatic analysis to predict the miRNAs with potential target sequences for binding to circ-Calm4. Furthermore, silencing circ-Calm4 increased the level of miR-124-3p upon hypoxia exposure, suggesting a negative correlation between circ-Calm4 and miR-124-3p expression and that circ-Calm4 acts as a sponge of miR-124-3p in PASMCs. Moreover, we confirmed interactions between and colocalization of circ-Calm4 and miR-124-3p. We further proved that circ-Calm4 mediated PASMC pyroptosis through miR-124-3p. These results indicate that circ-Calm4 regulates miR-124-3p by acting as a circRNA sponge via the ceRNA mechanism.

*Pdcd6* was originally known as apoptosis-linked gene and belongs to a Ca2+-binding protein family with EF-hand motifs.^43^ There is also evidence that the need of *Pdcd6* for apoptosis was questioned.^44^ Our study showed that the levels of apoptotic cells were decreased exposed to hypoxia, and these effects were significantly reversed by *Pdcd6* siRNA, suggested that *Pdcd6* involved in hypoxia-induced PASMCs apoptosis. However, whether *Pdcd6* is involved in pyroptosis has not been reported, the new function of *Pdcd6* need to be found. *Pdcd6* is reported to be a direct target gene of miR-124-3p in breast cancer cells,^34^ but this relationship has not been reported in PH. Our present study revealed that *Pdcd6*, which plays a crucial role in hypoxia-induced pyroptosis, was upregulated in hypoxia-exposed mice and in cellular models. In addition, our results demonstrated that changes in the circ-Calm4 level are associated with the expression level of *Pdcd6* and that knockdown of circ-Calm4 by sh-circ-Calm4 reverses the hypoxia-induced upregulation of *Pdcd6* in mice and cells. Furthermore, we demonstrated that circ-Calm4 modulates the expression of *Pdcd6* through miR-124-3p in PASMCs. More importantly, we proved that circ-Calm4 regulates PASMC pyroptosis via the miR-124-3p/*Pdcd6* axis in hypoxic PH. The findings revealed a new signaling pathway of pyroptosis mediated by circ-Calm4.

Pyroptosis is one form of programmed cell death. Similar to apoptosis, pyroptosis is mediated by the caspase family of cysteine proteases and is usually associated with the secretion of proinflammatory cytokines such as IL-1β and IL-18.^45^ In apoptosis, caspases cleave substrate proteins such as Caspase-3, -6, and -7, which in turn cleave specific target sites in many proteins, and leading to functional changes that result in the formation of apoptotic bodies.^46^ Like apoptotic bodies, pyroptotic bodies have a diameter of 1 to 5 µm.^47^ Morphologically, pyroptotic bodies can be categorized as an intermediate class between classic apoptotic bodies and apoptopodia. In our model, the annexin V-FITC/PI double staining results showed that pyroptotic cell death was upregulated in PASMCs exposed to hypoxia for 24 hours (quadrant 1), while apoptotic cell death was downregulated under exposure to hypoxia for 24 hours (quadrants 2 and 3; Figure 1F and Figure 6I). We also used an LDH release assay and PI staining^48^ to discriminate apoptotic and pyroptotic cells and found that PI staining and LDH activity were increased under hypoxia exposure. Taken together, our results indicated that pyroptosis and apoptosis play different roles in the pathogenesis of PH. However, the different roles of pyroptosis and apoptosis in PH need further exploration.

The hypothesis of ceRNA was first proposed by a research group of Pier Paolo Pandolfi of Harvard Medical School in 2011.^49^ It is a mode of gene expression regulation, the ceRNA hypothesis proposes that specific RNAs with shared microRNA (miRNA) binding sites compete for posttranscriptional control.^50^ An important factor in assessing the potential impact of the ceRNA hypothesis is that the ceRNA abundance must approach the miRNA target abundance to de-repress the miRNA targets.^51^ Our results are consistent with the reports, circ-Calm4 was present at roughly equal copy numbers as the mir-124-3p, which suggested the circ-Calm4 has a physiological role. The effect of miRNA-binding sites in circRNAs on ceRNA mechanism is still controversial.^50^ Actually, the new rigorous investigations are needed to be found. We performed pull down assays to demonstrate endogenous circ-Calm4 and miR-124-3p microRNA binding in vivo and in vitro, biotin-labeled circ-Calm4-specific probe and negative control probe were used to pull down the RNA complex, the synthetic 5′-end or 3′-end biotin-labeled DNA probes, which can bind to specific circRNA.^52^ However, the pulldown-probe is relatively long, and it is possible to interact with lincalm4 to some extent. Rigorous future studies are needed to delineate the sponge-effect of circRNAs. In our results, endogenous circ-Calm4 pull-down by AGO2 was only specifically enriched in miR-124-3p-treated cells and only after circCalm4 overexpression the interaction of AGO2 and miR-124-3p is detectable, these results partly validated that miR-124-3p was a circ-Calm4-targeted microRNA, but a stable AGO2 interaction is not seen at endogenous level. The possible explanation for this observation is mir-124-3p expression was decreased in hypoxic PASMCs. In our case, transfecting lincalm4 did not alter the expression of miR-124-3p in hypoxic PASMCs (Figure IVC in the Data Supplement), this finding illustrate that lincalm4 did not induce miR-124-3p degradation. However, that role of the lincalm4 released remains to be clarified, and whether circ-Calm4 playing the sole role on pyroptosis via sponge activity is still not completely clear. Apparently, more rigorous investigations are needed to solve this issue, and this is a limitation of the present study.

In summary, we showed that circ-Calm4 plays an important role in hypoxia-induced pyroptosis and is dependent on the circ-Calm4/miR-124-3p/*Pdcd6* axis. These findings provide a new strategy for understanding pathological alterations in PH and potential therapeutic targets for its treatment.

## Acknowledgment

We gratefully acknowledge the assistance of C. Jiang for the expert proofreading of our article.

## Sources of Funding

This work was supported by the National Natural Science Foundation of China (contract grant number 31820103007, 31971057, 31771276 to D. Zhu; and 81570041, 81870043 to B. Wu).

## Disclosures

None.

## Supplementary Material


