# Supplementary material for: Circular RNA Calm4 Regulates Hypoxia-Induced Pulmonary Arterial Smooth Muscle Cells Pyroptosis via the Circ-Calm4/miR-124-3p/PDCD6 Axis
Source: Arterioscler Thromb Vasc Biol. 2021 Mar 4;41(5):1675–93. doi: 10.1161/ATVBAHA.120.315525 (PMC8057524; doi:10.1161/ATVBAHA.120.315525)
Supplement: Supplementary file 1 [file atv-41-1675-s001.pdf]

Circular RNA Calm4 regulates hypoxia-induced pulmonary arterial smooth muscle cells pyroptosis via the circ-Calm4/miR-124-3p/PDCD6 axis

Running Title: Circ-RNA Calm-4 in hypoxia-induced PASMCs Pyroptosis

Yuan Jiang[1,2]*, Huiyu Liu[1,2]*, Hang Yu[1,2], Yang Zhou[3], Junting Zhang[1,2], Wei Xin[1,2], Yiying Li[1,2], Siyu He[1,2], Cui Ma[1,2], Xiaodong Zheng[1,2], Lixin Zhang[1,2], Xijuan Zhao[1,2], Bingxiang Wu[4], Chun Jiang[5], Daling Zhu[1,2,6,7]

[1]Central Laboratory of Harbin Medical University (Daqing), Daqing 163319, PR China
[2]College of Pharmacy, Harbin Medical University, Harbin 150081, PR China
[3]Department of MedicalOncology, The Third Affiliated Hospital of Harbin Medical U niversity, Harbin 150081, China
[4]The Second Affiliated Hospital of Harbin Medical University, No.246 Xuefu Road, Harbin, Heilongjiang Province, 150081, People's Republic of China.
[5]Department of Biology, Georgia State University, 50 Decatur Street, Atlanta, GA 30302, United States.
[6]State Province Key Laboratories of Biomedicine-Pharmaceutics of China, Daqing 163319, PR China
[7]Key Laboratory of Cardiovascular Medicine Research, Ministry of Education, Harbin Medical University, Harbin 150081, PR China
*These authors contributed equally to this work.
Corresponding author: College of Pharmacy, Harbin Medical University (Daqing), Xinyang Road, Daqing, Heilongjiang 163319, PR China.
E-mail addresses: dalingz@yahoo.com, zhudaling@hmudq.edu.cn (D. Zhu).

Supplementary Material

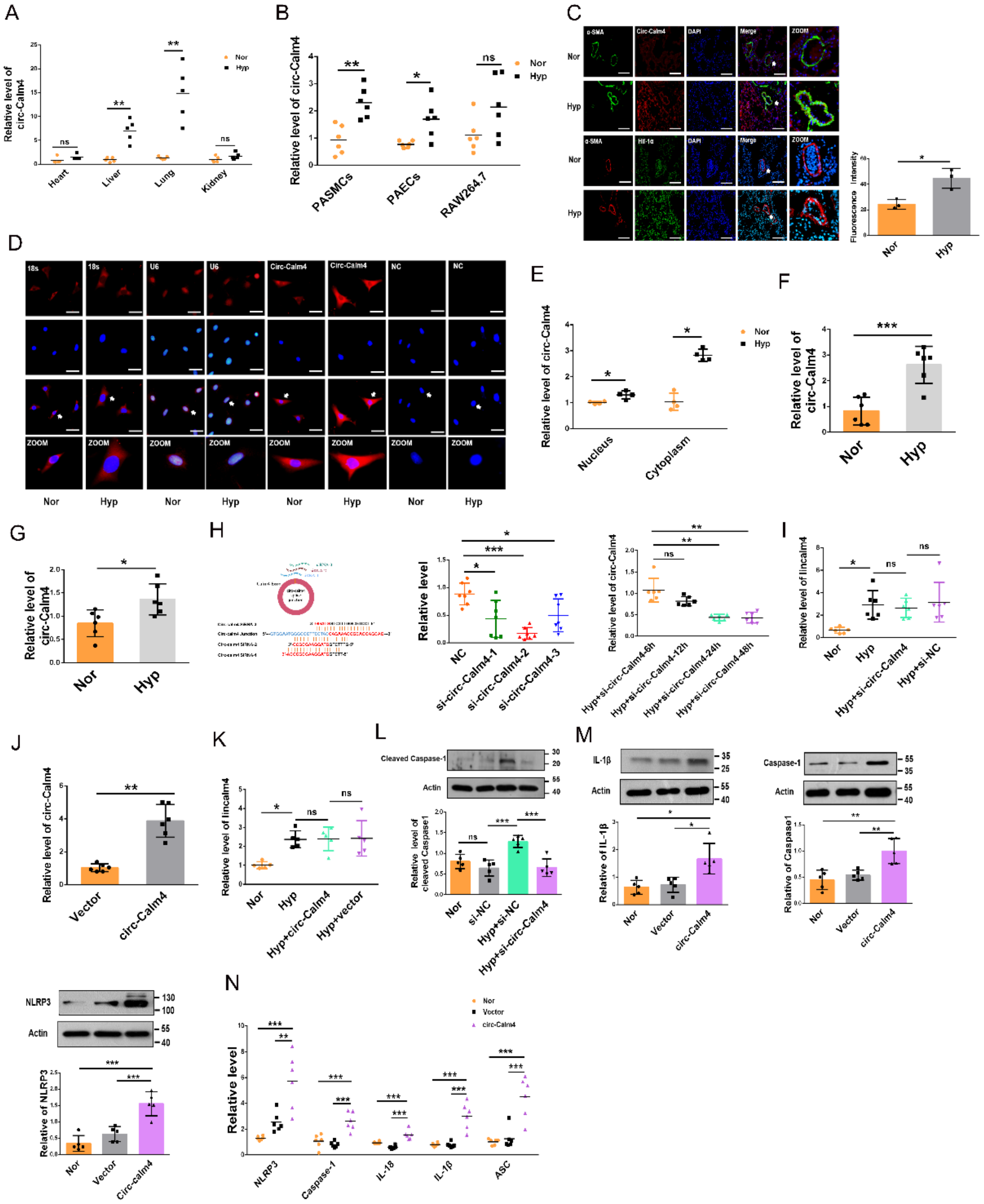

Figure I. Levels of circ-Calm4 in response to hypoxia.

A&B. The expression of circ-Calm4 in different tissues and cells. (n=5-6) **C.** Relative fluorescence of circ-Calm4 in mice exposed to hypoxia. Scale bars=100 µm. Circ-Calm4 probes were labeled with Cy3 (red). Nuclei were stained with DAPI (blue), pulmonary smooth muscle stained with α-SMA (green) and HIF-1α was used as positive control. (n=3) **D.** FISH showing the distribution of circ-Calm4 in the nucleus and cytoplasm. Scale bars=100 µm. Circ-calm4 probes were labeled with Cy3 (red). Nuclei were stained with DAPI (blue). U6 and 18S RNA were labeled with Cy3 (red) and NC-probe was used as a control. **E.** The mRNA levels of circ-Calm4 in the nuclear and cytoplasmic fractions upon hypoxia. (n=4). **F&G.** The expression of circ-Calm4 in

mice and medium from PASMCs. (n=6). **H.** Verification of the efficacy of circ-Calm4 in knocking down endogenous circ-Calm4 in PASMCs relative to that of the scramble negative control construct circ-Calm4 (n=6-7). **I**. Transfecting si-circ-Calm4-2 did not alter the expression of lincalm4 in PASMCs exposed to hypoxia. (n=6). **J.** Verification of circ-Calm4 overexpression at the mRNA level in PASMCs. (n=6). **K.** Circ-Calm4 overexpression did not alter the expression of lincalm4 in PASMCs exposed to hypoxia as detected by quantitative PCR (n=5). Circ-Calm4 siRNA reversed the increased protein levels of cleaved Caspase-1 induced by hypoxia in PASMCs (n=5). **L.** Circ-Calm4 siRNA reversed the increased protein levels of cleaved Caspase-1 induced by hypoxia in PASMCs (n=5). **M&N.** Circ-Calm4 overexpression increased the protein levels of NLRP3, Caspase-1, IL-1β and mRNA levels of *Nlrp3, Caspase-1, Il-1β, Il-18,* and *Asc* in PASMCs, which were detected by qPCR and western blot analysis, respectively. (n=4-6). Each datapoint in the figure represents a unique biological replicate. The data are presented as the means ± SD. Statistical analysis was performed with one-way ANOVA followed by Bonferroni correction and Student's t test for two means. The graph A, B and E were analyzed by the Mann Whitney U test. The graph H, I and M-IL-1β were analyzed by the Kruskal-Wallis test followed by Dunn post-test. *p <0.05, **p<0.01, ***p<0.001.

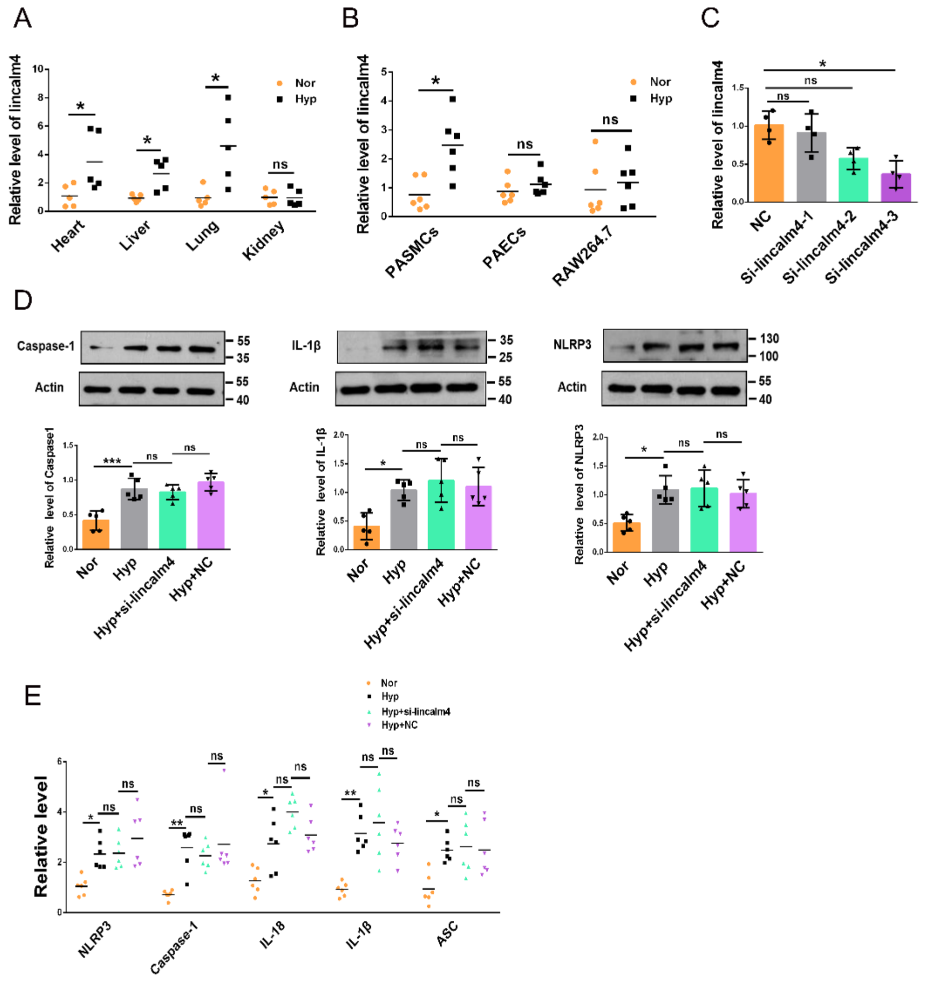

Figure II. Roles of lincalm4 in PASMC pyroptosis.

A&B. The expression of lincalm4 in different tissues and cells. (n=5-6). **C.** Verification of the efficacy of lincalm4 in knocking down endogenous lincalm4 in PASMCs. (n=4) **D&E.** Lincalm4 siRNA did not alter the protein levels of NLRP3, Caspase-1, IL-1β and mRNA levels of *Nlrp3, Caspase-1, Il-1β , Il-18,*and *Asc* in PASMCs exposed to hypoxia, which were detected by qPCR and western blot analysis, respectively. (n=4-6). Each datapoint in the figure represents a unique biological replicate. The data are presented as the means ± SD. Statistical analysis was performed with one-way ANOVA followed by Bonferroni correction and Student's t test for two means. The graph A and B were analyzed by the Mann Whitney U test, the graph C was analyzed by the Kruskal-Wallis test followed by Dunn post-test. *p <0.05, **p<0.01.

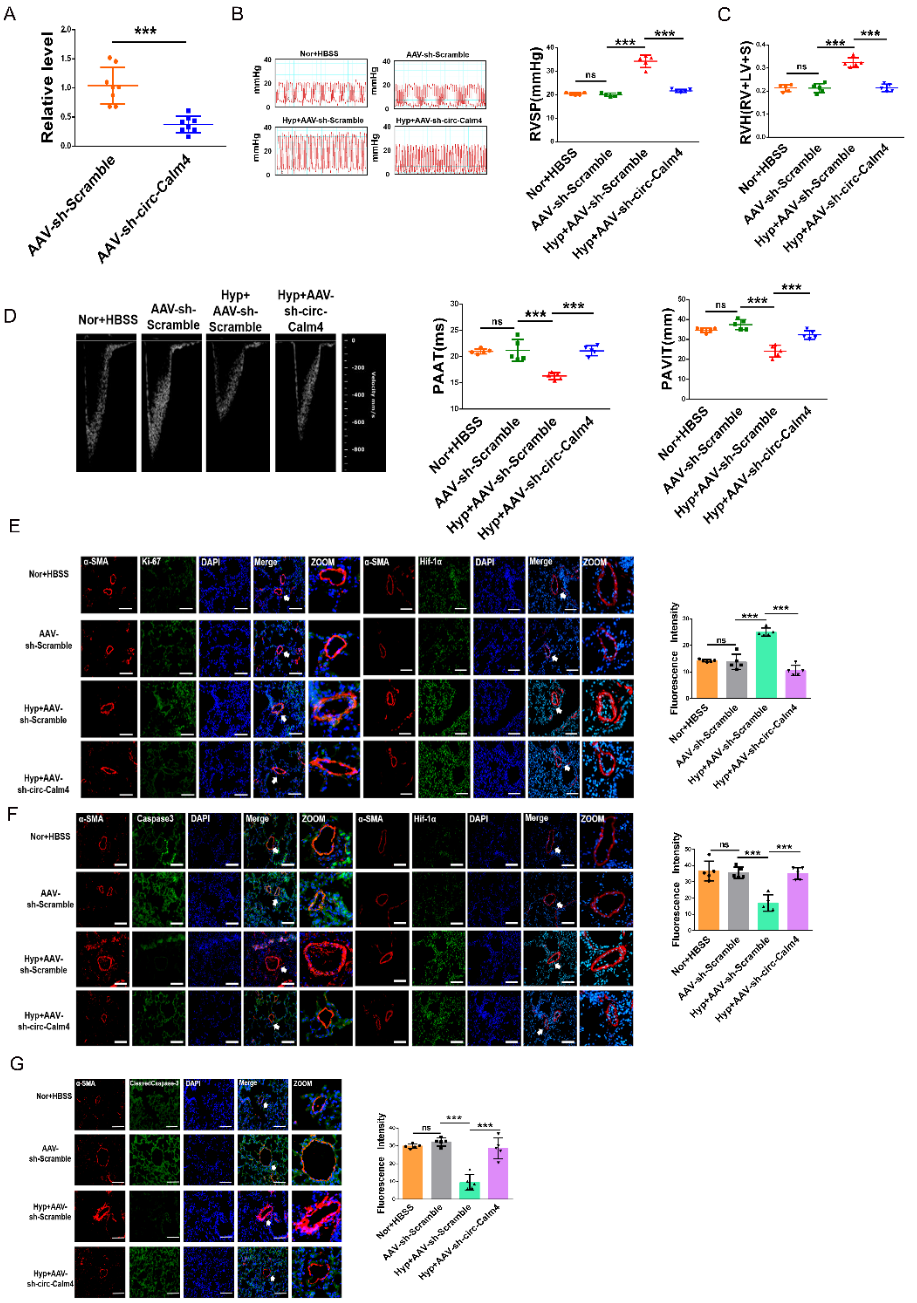

Figure III. Effects of circ-Calm4 on echocardiography.

A. Mice inhaled AAV9 viral particles carrying shRNA targeting circ-calm4 (sh-circ-calm4) or a scrambled control RNA fragment (sh-Scr) via dropwise intranasal instillation for ten days before establishment of the hypoxia-induced PH model. Specific knockdown of circ-Calm4 by sh-circ-Calm4 in mouse lung tissues was verified (n=8). **B & C.** The RVSP and RVH index values were lower in hypoxic mice with circ-Calm4 silencing via circ-Calm4 shRNA than in their counterparts (n=5). **D.** Echocardiographic image showing the increases in PAVTI and PAAT in hypoxic mice infected with AAV-sh-calm4 compared with hypoxic mice infected with NC-AAV-sh-calm4 (n=5). **E.** Knockdown of circ-Calm4 by sh-circ-Calm4 reversed the hypoxia-induced upregulation of Ki67 in mouse lung tissues. Scale bars=100 µm. Lung sections stained with Ki67 (green), pulmonary smooth muscle stained with α-SMA (red), DAPI (blue) for nuclear staining and HIF-1α (green) was used as positive control. **F.** Knockdown of circ-Calm4 by sh-circ-Calm4 increased the expression of Caspase3 in mouse lung tissues upon hypoxia. Scale bars=100 µm. Lung sections stained with Caspase3(green), pulmonary smooth muscle stained with α-SMA (red), DAPI (blue) for nuclear staining and HIF-1α (green) was used as positive control. **G.** Knockdown of circ-Calm4 by sh-circ-Calm4 increased the expression of cleaved-Caspase3 in mouse lung tissues upon hypoxia. Scale bars=100 µm. Lung sections stained with cleaved-Caspase3 (green), pulmonary smooth muscle stained with α-SMA (red), DAPI for nuclear staining. Each datapoint in the figure represents a unique biological replicate. The data are presented as the means ± SD. Statistical analysis was performed with one-way ANOVA followed by Bonferroni correction and Student's t test for two means. *p <0.05, **p<0.01, ***p<0.001.

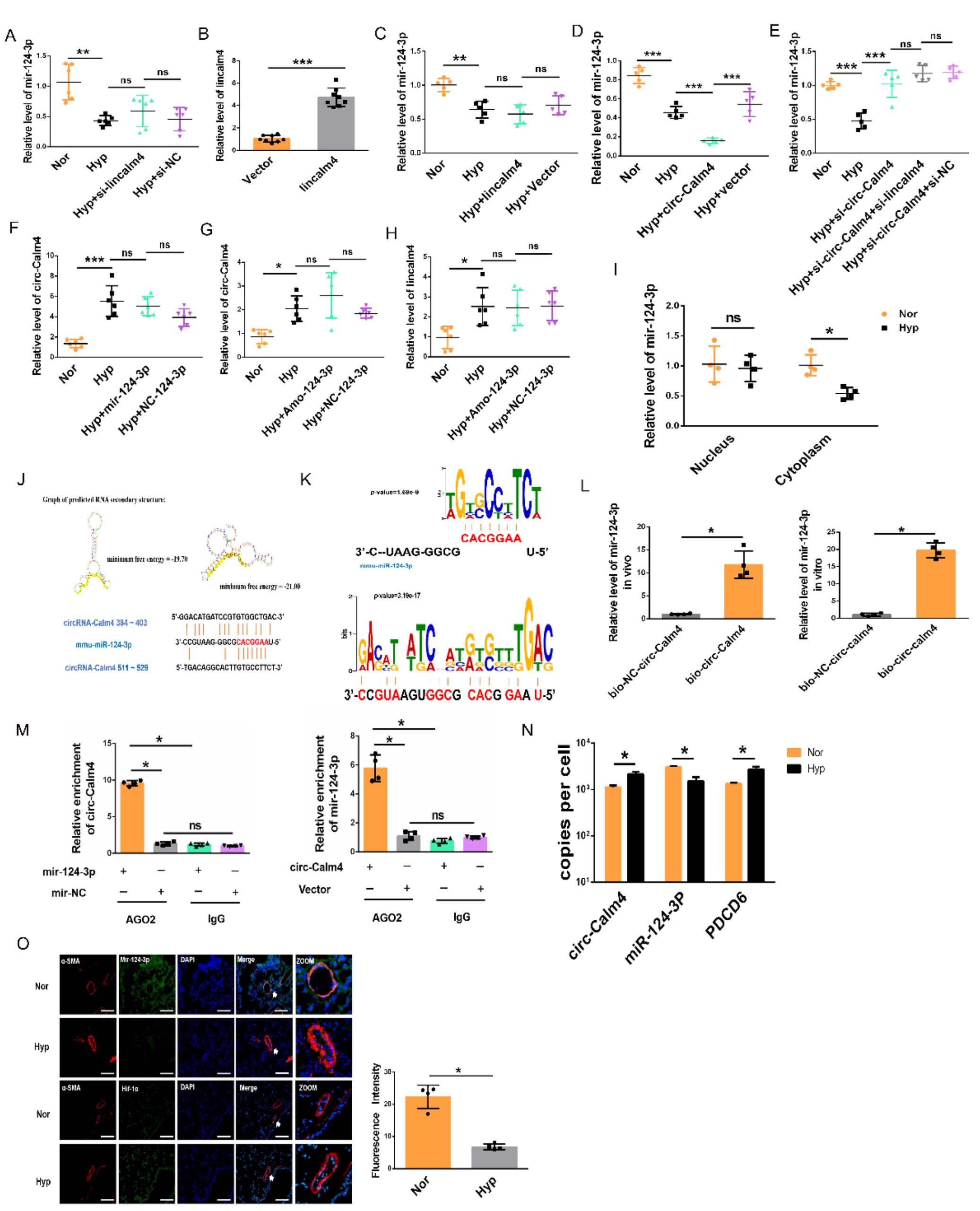

Figure IV. MiR-124-3p is a downstream target gene of circ-Calm4.

A. Knockdown of lincalm4 by siRNA did not alter the expression of mir-124-3p in PASMCs exposed to hypoxia as detected by quantitative PCR (n=6). **B.** Verification of lincalm4 overexpression at the mRNA level in PASMCs. (n=8) **C**. Overexpression of lincalm4 did not alter the expression of mir-124-3p in PASMCs exposed to hypoxia. (n=5); **D.** Overexpression of circ-Calm4 resulted in down-regulation of mir-124-3p in hypoxic PASMCs as detected by quantitative PCR (n=6). **E.** Circ-Calm4-siRNA abrogated the hypoxia-induced expression downregulation of mir-124-3p at mRNA level, whereas cotransfection with circ-Calm4 siRNA and lincalm4 siRNA did not alter the expression of miR-124-3p upon hypoxia exposure, as indicated by quantitative PCR (n=5). **F.** Transfecting miR-124-3p mimic did not altered the expression of circ-Calm4 in hypoxic PASMCs as detected by quantitative PCR (n=6). **G.** Transfecting AMO-124-3p did not altered the expression of circ-Calm4 in hypoxic PASMCs as detected by

quantitative PCR (n=6). **H.** AMO-124-3p did not alter the expression of lincalm4 in hypoxic PASMCs as detected by quantitative PCR (n=6). **I**. The mRNA levels of miR-124-3p in the nuclear and cytoplasmic fractions upon hypoxia. (n=4). **J.** Complementary sequences of circ-Calm4 with miR-124-3p is shown. **K.** We used the position p-value to describe the potential of base pairing, the computational motif analysis of circ-Calm4 were p-value =1.69e-9 and p-value =3.19e-17 of the two miR-124-3p targets sites. The position p-value is defined as the probability that a random sequence would have a match to the motif under test with a score greater or equal to the largest found in the sequence under test.The combined match p-value is defined as the probability that a random sequence would have position p-values such that the product is smaller or equal to the value calculated for the sequence under test. The combined match p-value of base pairing is 6.42e-29. The E-value of a motif is based on its log likelihood ratio, width, sites, the background letter frequencies and the size of the training set. The MEME's e-value of the targets sites is 1.9e-003. **L.** MiR-124-3p was pulled down from hypoxia-induced PH mice model and hypoxia-exposed PASMCs lysate by biotin-labeled circ-Calm4 probe, the level of miR-124-3p was detected by quantitative PCR (n=4). **M.** AGO2 protein-based RNA immunoprecipitation assay for the association between circ-Calm4 and miR-124-3p, the level of circ-Calm4 and miR-124-3p were detected by quantitative PCR (n=4). **N.** Absolute copies per cell of circ-Calm4, miR-124-3p and *Pdcd6* were detected by quantitative PCR (n=4). **O.** Downregulation of miR-124-3p in mice. Scale bars=100 µm. Lung sections were stained for miR-124-3p probes (green), pulmonary smooth muscle was stained for α-SMA (red), DAPI for nuclear staining and *Hif-1α* was used as positive control. Each datapoint in the figure represents a unique biological replicate. The data are presented as the means ± SD. Statistical analysis was performed with one-way ANOVA followed by Bonferroni correction and Student’s t test for two means. The graph A was analyzed by the Kruskal-Wallis test followed by Dunn post-test. The graph L, M and O were analyzed by the Mann Whitney U test. *p <0.05, **p<0.01, ***p<0.001.

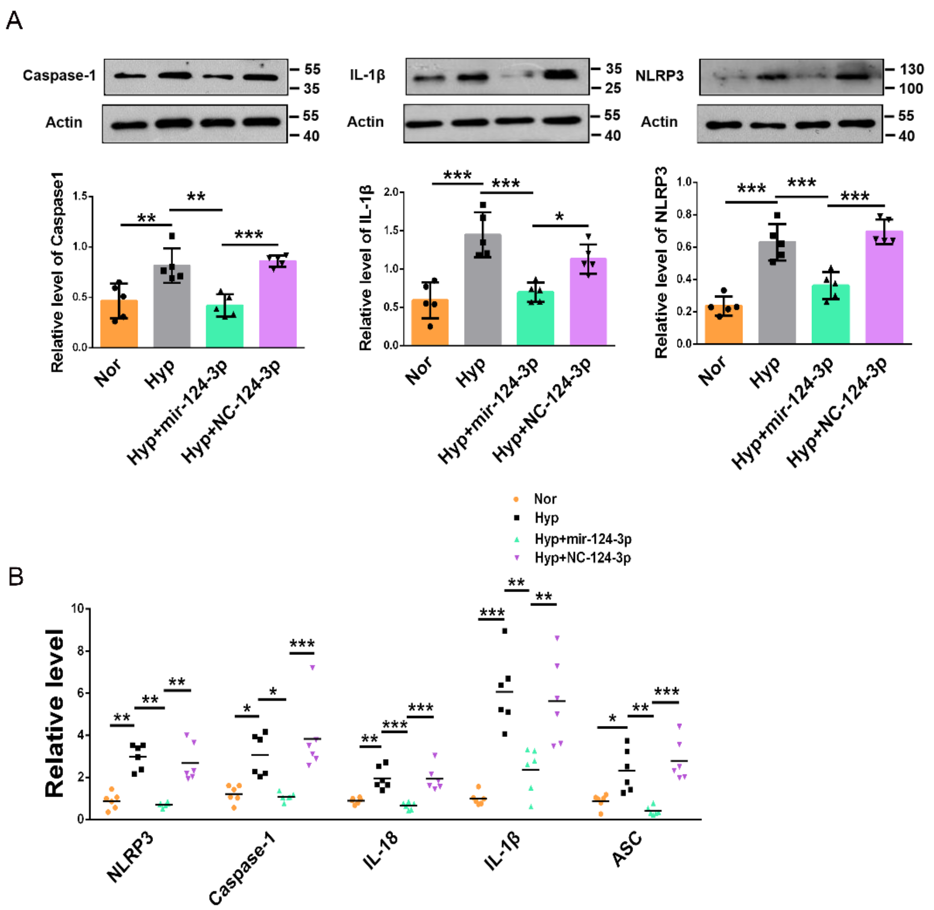

Figure V. Mir-124-3p inhibits pyroptosis in PASMCs.

A & B. In PASMCs exposed to hypoxia, transfection of the miR-124-3p mimic decreased the protein levels of NLRP3, Caspase-1, IL-1β and mRNA levels of *Nlrp3, Caspase-1, Il-1β, Il-18,* and *Asc*, which were detected by qPCR and western blot analysis, respectively. (n=3-6). Each datapoint in the figure represents a unique biological replicate. The data are presented as the means ± SD. Statistical analysis was performed with one-way ANOVA followed by Bonferroni correction. *p <0.05, **p<0.01. ***p<0.001.

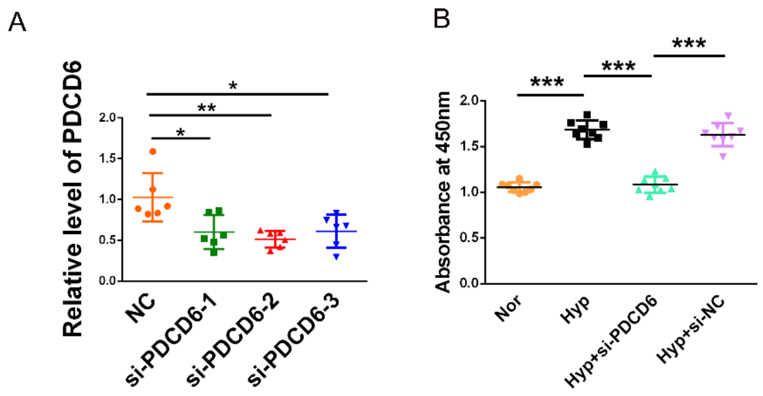

Figure VI. Transfection of PDCD6.

A. The efficacy of si-PDCD6 in knocking down endogenous *Pdcd6* in PASMCs relative to that of the negative control construct NC-si-PDCD6 (n=6). **B.** Knockdown of endogenous *Pdcd6* abrogated the increasing proliferation of PASMCs induced by hypoxia. The survival rate was studied by CCK8 assay. (n=8). Each datapoint in the figure represents a unique biological replicate. The data are presented as the means ± SD. Statistical analysis was performed with one-way ANOVA followed by Bonferroni correction. **p* <0.05*,* ***p*<0.01. ****p*<0.001.

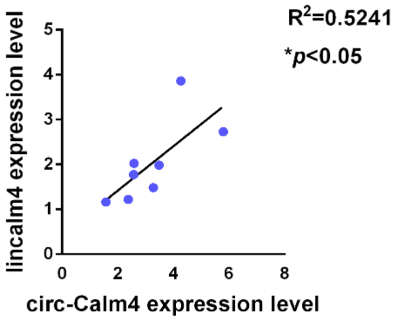

Figure VII. Correlation analysis of circ-Calm4 and lincalm4

Circ-calm4 expression was positively correlated with lincalm4 expression in hypoxic mice. Statistical analysis was performed with Pearson's correlation test. **p* <0.05.

Major Resources Table

In order to allow validation and replication of experiments, all essential research materials listed in the Methods should be included in the Major Resources Table below. Authors are encouraged to use public repositories for protocols, data, code, and other materials and provide persistent identifiers and/or links to repositories when available. Authors may add or delete rows as needed.

Animals (in vivo studies)

Species	Vendor or Source	Background Strain	Sex	Persistent ID / URL
mouse	Harbin Medical University	C57BL/6J	male	

Antibodies

Target antigen	Vendor or Source	Catalog #	Working concentration	Lot # (preferred but not required)	Persistent ID / URL
Caspase-1	BOSTER	BA2220	1ug/ml		http://www.boster.com.cn/index/index.html
NLRP3	BOSTER	BA3677	1ug/ml		http://www.boster.com.cn/index/index.html
IL-1β	BIOSS	bs-10859R	2ug/ml		http://www.bioss.com.cn/index.asp
ASC	BIOSS	bs-6741R	2ug/ml		http://www.bioss.com.cn/index.asp
IL-18	Proteintech	10663-1-AP	0.76ug/ml		http://www.ptgcn.com/
PDCD6	Proteintech	12303-1-AP	0.734ug/ml		http://www.ptgcn.com/
Caspase-3	Proteintech	19677-1-AP	5.4ug/ml		http://www.ptgcn.com/
Ki-67	BOSTER	bs-2130R	10ug/ml		http://www.boster.com.cn/index/index.html
CleavedCaspase-3	Cell Signaling Technology	#9661	5ug/ml		https://www.cellsignal.cn/
Cleaved Caspase-1	Cell Signaling Technology	#89332	1ug/ml		https://www.cellsignal.cn/
α-SMA	Proteintech	14395-1-AP	4ug/ml		http://www.ptgcn.com/

DNA/cDNA Clones

Clone Name	Sequence	Source / Repository	Persistent ID / URL
mmu-miR-124-3p	5′-UAAGGCACGCGGUGAAUGCC-3′ 5′-CAUUCACCGCGUGCCUUAUU-3′	GeneChem	https://www.genechem.com.cn/
mmu-AMO-124-3p	5′-GGCAUUCACCGCGUGCCUUA-3′.	GeneChem	https://www.genechem.com.cn/
mmu-miR-NC	5′-UUCUCCGAACGUGUCACGUTT-3′ 5′-ACGUGACACGUUCGGAGAATT-3′	GeneChem	https://www.genechem.com.cn/
mmu-AMO-NC	5′-CAGUACUUUUGUGUAGUACAA-3′	GeneChem	https://www.genechem.com.cn/
Si-Circ-calm4-1 target	5′-TGGGCGCTTCCTACCAGAA-3′	RIBOBIO	http://ribobio.bioon.com.cn/
Si-Circ-calm4-2 target	5′-GGCGCTTCCTACCAGAAAC-3′	RIBOBIO	http://ribobio.bioon.com.cn/
Si-Circ-calm4-3 target	5′-CCTACCAGAAACCGCAGGA-3′	RIBOBIO	http://ribobio.bioon.com.cn/
Si-PDCD6-1 target	5′-GCTTCCTGTGGAACGTCTT-3′	RIBOBIO	http://ribobio.bioon.com.cn/
Si-PDCD6-2 target	5′-GGGACAACTCTGGGATGAT-3′	RIBOBIO	http://ribobio.bioon.com.cn/
Si-PDCD6-3 target	5′-GGATCAGGATGGCTGGATT-3′	RIBOBIO	http://ribobio.bioon.com.cn/

Cultured Cells

Name	Vendor or Source	Sex (F, M, or unknown)	Persistent ID / URL
PASMCs	Mouse from Harbin Medical University	M	

Data & Code Availability

Description	Source / Repository	Persistent ID / URL
Data available on request from the authors	the authors	

Other

Description	Source / Repository	Persistent ID / URL
Dual Luciferase Reporter Assay	Beyotime Biotechnology	https://www.beyotime.com/index.htm
Fluorescence In Situ Hybridization Kit	GeneChem	https://www.genechem.com.cn/
Annexin V-FITC/PI Kit	Beyotime Biotechnology	https://www.beyotime.com/index.htm
Hoechst 33342/PI fluorescence staining Kit	Beyotime Biotechnology	https://www.beyotime.com/index.htm
LDH release agent	Beyotime Biotechnology	https://www.beyotime.com/index.htm
CCK8 assay	Beyotime Biotechnology	https://www.beyotime.com/index.htm
RNA Antisense Purification (RAP) Kit	BersinBioTM	http://biosense.bioon.com.cn/
RNA Immunoprecipitation (RIP) Kit	BersinBioTM	http://biosense.bioon.com.cn/
