# Supplementary figures and images for: Circular RNA Calm4 Regulates Hypoxia-Induced Pulmonary Arterial Smooth Muscle Cells Pyroptosis via the Circ-Calm4/miR-124-3p/PDCD6 Axis
Source: Arterioscler Thromb Vasc Biol. 2021 Mar 4;41(5):1675–93. doi: 10.1161/ATVBAHA.120.315525 (PMC8057524; doi:10.1161/ATVBAHA.120.315525)

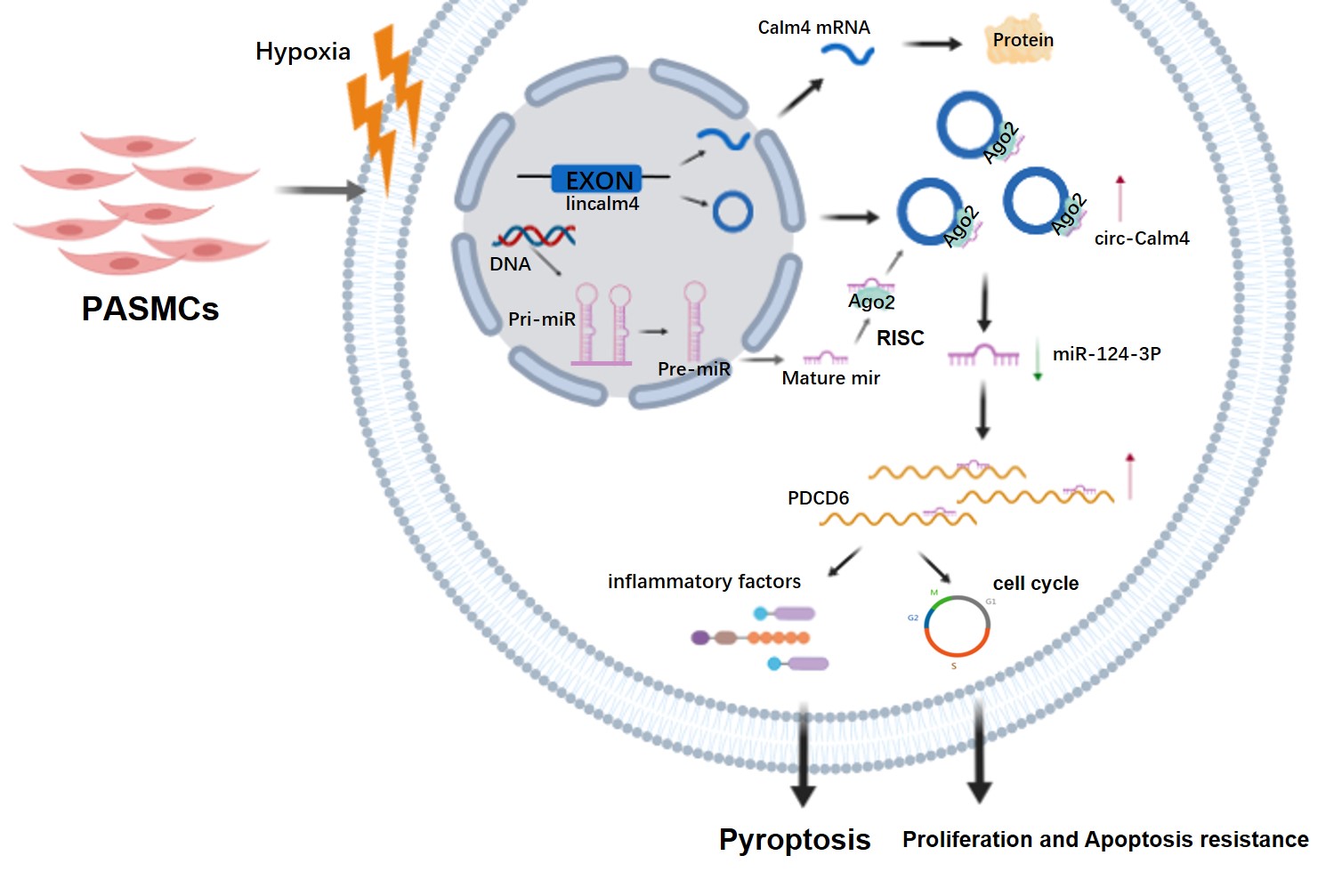

Supplement: Supplementary file 2 [file atv-41-1675-s002.jpg]
